# Nanoplastics Impair GnRH Neuron Migration and Neuroendocrine Function: Emerging Players in the Pathogenesis of Reproductive Disorders

**DOI:** 10.1002/smll.202506171

**Published:** 2026-02-06

**Authors:** Federica Amoruso, Alyssa Julia Jennifer Paganoni, Astrid Saraceni, Andrea Magnani, Alessia Brossa, Giorgio Roberto Merlo, Cristina Matei, Ruben Heinjan Willemsen, Raíssa Carneiro Rezende, Alexander Augusto de Lima Jorge, Federica Dal Bello, Patrizia Bovolin, Sasha Rose Howard, Roberto Oleari, Anna Cariboni

**Affiliations:** ^1^ Department of Pharmacological and Biomolecular Sciences “Rodolfo Paoletti” Università degli Studi di Milano Milan Italy; ^2^ Department of Life Sciences and Systems Biology Università di Torino Turin Italy; ^3^ SUSPLAS@UniTo Sustainable Plastic Scientific Hub Università di Torino Turin Italy; ^4^ Department of Molecular Biotechnology and Science for Health Università di Torino Turin Italy; ^5^ Genetic Endocrinology Unit (LIM25) Endocrinology Division Faculdade De Medicina da Universidade de São Paulo (HC‐FMUSP) São Paulo Brazil; ^6^ Centre for Endocrinology William Harvey Research Institute Barts, and the London School of Medicine and Dentistry Queen Mary University of London London UK; ^7^ Department of Paediatric Endocrinology Barts Health NHS Trust London UK; ^8^ Lister Hospital East and North Hertfordshire Teaching NHS Trust Stevenage UK; ^9^ Coordinating Research Center on Nano/microplastics on Environments Medicine Ecology Systems Interaction Studies – CRC NEMESIS Università degli Studi di Milano Milan Italy

**Keywords:** GnRH deficiency, GnRH neurons, nanoplastics, neuroendocrine function, neuron migration

## Abstract

Nanoplastics (NPs) pose an emerging threat to environmental and human health. Still, the impacts of NPs on the endocrine control of reproduction remain poorly understood, despite increasing trends of infertility worldwide. In mammals, reproductive function relies on the hypothalamus‐pituitary‐gonadal axis, centrally regulated by gonadotropin‐releasing hormone (GnRH) neurons. Disruption in GnRH neuron development or function leads to GnRH deficiency (GD), a genetic condition presenting delayed puberty and infertility. Yet, genetic causes explain only ∼50% of GD cases, suggesting a role for environmental factors in disease etiology. Here, we investigate NP effects on GnRH neuron biology by applying two established in vitro models: hormone‐secreting GT1‐7 cells and migrating GN11 cells. We show that NPs enter cells via non‐classical endocytosis, alter neuroendocrine function in GT1‐7 cells, and impair migration in GN11 cells. Transcriptomic analysis of NP‐exposed GN11 cells reveals differential expression of key genes linked to GnRH neuron development. Moreover, integrating these findings with exome sequencing data from patients with GD identifies rare *NPAS2* variants in two males with severe pubertal delay. These results suggest that PS‐NPs disrupt key physiological functions of GnRH neurons and may act as novel endocrine disruptors, contributing to the pathogenesis of reproductive disorders.

## Graphical abstract

Little is known about the effects of polystyrene nanoplastics (PS‐NPs) on gonadotropin releasing hormone (GnRH) neurons, which control puberty onset and fertility. By applying tailored in vitro GnRH neuron models, this article shows how PS‐NPs are internalized and impact hormone secretion and cell migration, through gene expression changes. Ultimately, integration of transcriptomic and human clinical data pointed out NPAS2 as a possible new environmentally influenced gene in reproductive disorders.

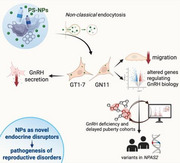

## 1 Introduction

Plastic pollution, especially from mismanaged waste degrading into nanoscale debris known as nanoplastics (NPs), has become a pervasive environmental issue raising significant concerns for human health [1, 2, 3]. NPs are usually composed of various polymers including polystyrene (PS), a widely used plastic produced by polymerizing styrene monomers [4]. These plastic particles infiltrate most ecosystems, enter the food chain, cross biological barriers including the blood brain barrier (BBB), and bioaccumulate in various human organs and tissues, comprising the brain [5, 6, 7]. Despite growing evidence of NP‐induced behavioral, metabolic, and developmental abnormalities, research on their influence on reproduction and fertility remains sparse [8].

Reproductive function in mammals relies on a functional hypothalamus‐pituitary‐gonad (HPG) axis, centrally regulated by gonadotropin‐releasing hormone (GnRH) neurons. During embryogenesis, GnRH neurons migrate from the olfactory placode into the hypothalamus, where they secrete GnRH hormone. Alterations in GnRH neuron migration or secretion may lead to GnRH deficiency (GD), with consequent reproductive failure and infertility. Numerous gene variants have been identified in patients with GD, however these account for approximately 50% of known cases, leaving a substantial proportion still unexplained [9, 10, 11]. This gap suggests that, in combination with genetic factors, environmental toxicants such as NPs may contribute to GD etiology, possibly by altering the expression of genes involved in GnRH neuron biology. Furthermore, epidemiological data highlight alarming trends on declines in global fertility rates and difficulties in natural conception [12, 13]. While these patterns are influenced by multiple factors, some studies have already explored the effects of environmental endocrine disruptors on reproductive function in both sexes [14, 15]. In this context, NPs may represent potential environmental toxicants contributing to adverse fertility outcomes [16]. Indeed, NPs are known to impact GnRH secretion in adult female rats and zebrafish, although the underlying cellular and molecular mechanisms remain unknown [17, 18]. Furthermore, and supporting a potential effect of NPs on the early development of the HPG axis, these plastic particles can cross the placenta and reach the embryo [5, 19, 20]. Still, their specific impact on the development of embryonic GnRH neurons has not been investigated yet.

In this study, by applying established in vitro models of mature hormone‐secreting (GT1‐7) and immature migrating (GN11) GnRH neurons, we provide evidence that polystyrene NPs (PS‐NPs) are internalized by both cell types via a mechanism that involves clathrin‐ and caveolin‐independent internalization. We further demonstrate that PS‐NP exposure impacts neurohormone release and cell migration, altering key cellular functions and transcriptional programs essential for early HPG axis development. Last, to explore potential clinical relevance, we integrated transcriptomic data of PS‐NP‐treated GnRH neurons with genome‐wide association studies (GWAS) of pubertal timing and exome sequencing from GD patients. This analysis revealed several genes that are both influenced by PS‐NP exposure and implicated in the regulation of pubertal timing, including a novel candidate gene found mutated in two patients with GD.

Together, our findings provide first insights into how PS‐NP pollution can trigger critical changes in hypothalamic GnRH neurons and may contribute to the molecular etiology of reproductive disorders via gene‐environment interactions.

## 2 Results

### 2.1 PS‐NPs Enter GT1‐7 Cells Via Non‐Classical Endocytosis and Impact Neuroendocrine Function

To understand the mechanisms through which NPs can alter GnRH secretion in vivo, we utilized an immortalized GnRH neuron murine cell line, GT1‐7, as a well‐established model of maturing GnRH neurons able to secrete GnRH in culture [17, 18, 21].

For all the experiments, commercially available PS‐NPs with a green, fluorescent dye embedded in the polymer matrix were chosen. At first, we assessed whether PS‐NPs of 500 nm diameter exerted any cytotoxic effects on the cells. GT1‐7 cells were therefore exposed to PS‐NPs at concentrations ranging from 50 to 200 µg/mL, based on concentrations already established in previous studies on neuronal in vitro models [22]. Cell viability was assessed by MTT assays after 24 and 48 h (h). Our results showed no significant differences in cell viability between NP‐exposed and unexposed samples across all concentrations and time points (Figure S1).

**FIGURE 1:**
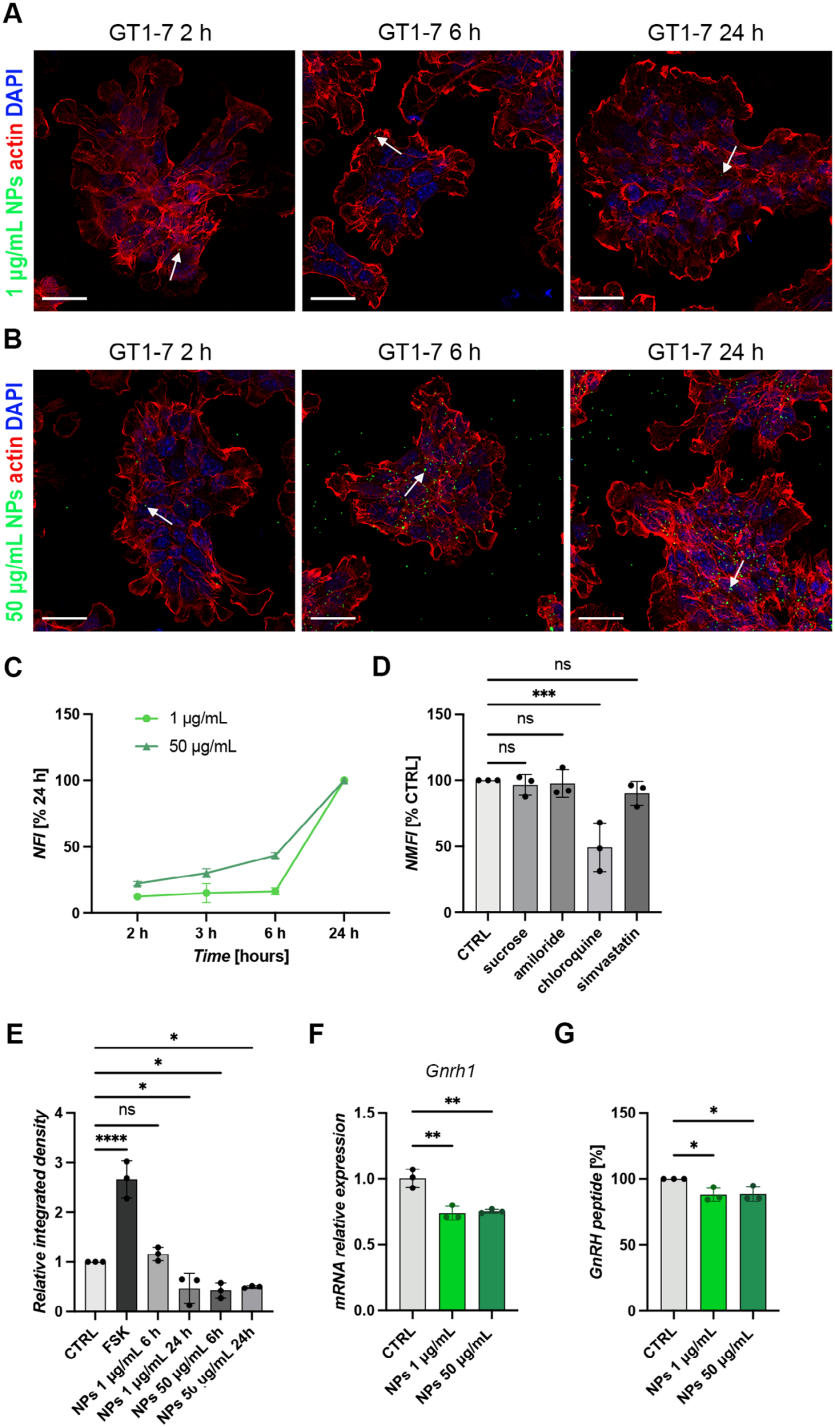
Non‐toxic concentrations of PS‐NPs are internalized into GT1‐7 cells by clathrin and caveolin‐independent endocytosis and impact GnRH hormone production. (A‐B) Confocal microscopy images of GT1‐7 cells treated with 1 µg/mL (A) 50 µg/mL (B) fluorescent PS‐NPs for 2, 6, and 24 h. PS‐NPs (green) are internalized and localize in the cytoplasm (arrows). Nuclei are stained with DAPI (blue) and the actin cytoskeleton with phalloidin (red). Scale bar: 50 µm. (C) Flow cytometry analysis of PS‐NP uptake inside GT1‐7 cells treated with 1 (light green) and 50 µg/mL (dark green) concentrations for increasing durations (2, 3, 6, and 24 h). Data are shown as normalized fluorescence intensity (NFI, % of 24 h) as a function of PS‐NPs exposure time (*N* = 3). (D) NFI (% of untreated CTRL) in GT1‐7 cells exposed to 50 µg/mL PS‐NPs for 24 h following 1 h pre‐treatment with endocytosis inhibitors. GT1‐7 cells exposed to PS‐NPs but not pre‐treated with inhibitors were used as CTRL (*N* = 3; One Way ANOVA followed by Dunnett's multiple comparisons test, ^***^ *p* < 0.001, ns non significant; sucrose *p* = 0.9844, amiloride *p* = 0.9965, chloroquine *p* = 0.0007, simvastatin *p* = 0.6517). (E) Quantification of GnRH staining by integrated density analysis (*N* = 3; One Way ANOVA followed by Dunnett's multiple comparisons test, ^*^ *p* < 0.05; ^****^ *p* < 0.0001; FSK *p* < 0.0001, NPs 1 µg/mL 6 h *p* = 0.8259, NPs 50 µg/mL 24 h *p* = 0.0366, NPs 50 µg/mL 6 h *p* = 0.0205, NPs 50 µg/mL 24 h *p* = 0.0389). (F) qPCR analysis of GnRH mRNA expression in GT1‐7 cells treated with 1 and 50 µg/mL PS‐NPs for 24 h compared to untreated (CTRL) cells (*N* = 3; One Way ANOVA followed by Dunnett's multiple comparisons test, ^**^ *p* < 0.01; PS‐NPs 1 µg/mL *p* = 0.0013, PS‐NPs 50 µg/mL *p* = 0.0018). (G) GnRH peptide quantification following 24 h exposure to 1 and 50 µg/mL PS‐NPs compare with untreated CTRL (*N* = 3; One Way ANOVA followed by Dunnett's multiple comparisons test, ^*^ *p* < 0.05; PS‐NPs 1 µg/mL *p* = 0.0247, PS‐NPs 50 µg/mL *p* = 0.0291).

Next, to examine cellular internalization of PS‐NPs, cells were exposed to concentrations of 1 and 50 µg/mL for 2, 6, and 24 h. Direct fluorescence imaging using confocal microscopy was performed after cell fixation and nuclei/cytoskeleton staining. Imaging revealed initial internalization of NPs after 2 h of exposure, with uptake progressively increasing up to 24 h (Figure 1A,B).

To further validate cellular uptake of PS‐NPs, we quantified the fluorescence intensity of PS‐NP‐treated cells using flow cytometry. Figure 1C depicts the Normalized Fluorescence Intensity (NFI) as a function of exposure time for GT1‐7 cells treated with 1 and 50 µg/mL PS‐NPs. Consistent with confocal images, NPs are internalized by GT1‐7 cells, and the fluorescence intensity of PS‐NP‐treated cells increased over time.

Then, to identify the main mechanisms of internalization, cells were pre‐treated for 1 h with inhibitors targeting specific endocytic pathways before exposure to 50 µg/mL PS‐NPs. This concentration was selected based on evidence of a visible and consistent internalization of PS‐NPs compared to the lower 1 µg/mL dose at all the time points considered in previous analyses. Specific inhibitors and doses were used as follows: 45 mM sucrose to block the clathrin‐mediated pathway, 10 µg/mL amiloride hydrochloride to block micropinocytosis, 20 µM chloroquine to inhibit caveolin‐ and clathrin‐independent endocytosis, 2.5 µM simvastatin or 25 µg/mL nystatin to halt caveolae‐mediated pathway. Inhibitors and their doses were selected based on previous work [23]. Flow cytometry analysis demonstrated a significant decrease in PS‐NP internalization following chloroquine pre‐treatment, whereas no significant reduction was observed with sucrose, amiloride, or simvastatin pre‐treatment (Figure 1D; mean % to control (CTRL) ± SD: sucrose, 96.58 ± 7.77; amiloride, 97.70 ± 10.56; chloroquine, 49.36 ± 18.37; simvastatin, 90.20 ± 8.96). These data suggest that PS‐NP uptake primarily occurs through a clathrin‐ and caveolae‐independent endocytic mechanism.

To demonstrate a direct effect of PS‐NPs exposure on intracellular GnRH levels, GT1‐7 cells were exposed to 1 and 50 µg/mL PS‐NPs for 6 and 24 h, and GnRH content measured by immunostaining. Forskolin was included as a positive control, as previously reported [24, 25]. Quantitative analysis of GnRH immunoreactivity revealed a significant reduction in intracellular GnRH peptide in GT1‐7 cells treated with either PS‐NPs concentration compared to untreated cells, quantified by integrated density analysis (Figure S2 and Figure 1E; mean ratio to CTRL ± SD: FSK, 2.66 ± 0.37; NPs 1 µg/mL 6 h, 1.16 ± 0.14; NPs 1 µg/mL 24 h, 0.47 ± 0.30; NPs 50 µg/mL 6 h, 0.43 ± 0.15; NPs 50 µg/mL 24 h, 0.50 ± 0.02). These results indicate that exposure of GT1‐7 cells to non‐toxic PS‐NPs concentrations directly impairs intracellular levels of GnRH peptide.

We next investigated whether this decrease was associated with impaired transcriptional activity and/or increased peptide release. To this end, we quantified GnRH transcript expression and GnRH peptide secretion by quantitative PCR (qPCR) and high‐resolution mass spectrometry (HRMS), respectively. Specifically, GT1‐7 cells were treated with 1 and 50 µg/mL PS‐NPs for 24 h, the timepoint at which intracellular GnRH was significantly reduced. *Gnrh1* mRNA levels were significantly reduced in cells exposed to both PS‐NPs concentrations compared to untreated (CTRL) cells (Figure 1F; mean CTRL ± SD: CTRL, 1.01 ± 0.07; PS‐NPs 1 µg/mL, 0.74 ± 0.05; PS‐NPs 1 µg/mL, 0.75 ± 0.02). Consistently, nano High Performance Liquid Chromatography (HPLC)‐HRMS analysis of conditioned medium (CM) from GT1‐7 cells revealed a significant reduction in secreted GnRH following exposure to both concentrations of PS‐NPs (Figure 1G; Figure S2B; mean % to CTRL ± SD: PS‐NPs 1 µg/mL, 88.11 ± 4.97; PS‐NPs 1 µg/mL, 88.57 ± 5.43).

Together, these findings demonstrate that PS‐NPs lead to reduced neurohormone secretion of maturing GnRH neurons by a mechanism that affects GnRH gene expression.

### 2.2 Intracellular Uptake of PS‐NPs in GN11 Cells Occur Via Endocytic Pathways Independent of Clathrin and Caveolin

To investigate the possible impact of PS‐NPs during the development of GnRH neuroendocrine cells, we used the GN11 murine cell line as a model of developing GnRH neurons characterized by efficient migratory ability in vitro [24, 25, 26].

First, we assessed the ability of 500 nm diameter PS‐NPs to be internalized by GN11 by exposing cells to increasing concentrations of PS‐NPs (ranging from 50 ng/mL to 200 µg/mL), as per previous experiments in GT1‐7 cells. GN11 cell vitality upon PS‐NPs exposure was evaluated via MTT, and no significant differences between treated versus untreated cells were found after 24 h of exposure for all concentrations tested. In contrast, prolonged exposure (48 h) resulted in reduced GN11 cell vitality (Figure S3). This might be explained by a higher sensitivity of immature GN11 cells to prolonged PS‐NP exposure, compared to mature GT1‐7 cells.

**FIGURE 2:**
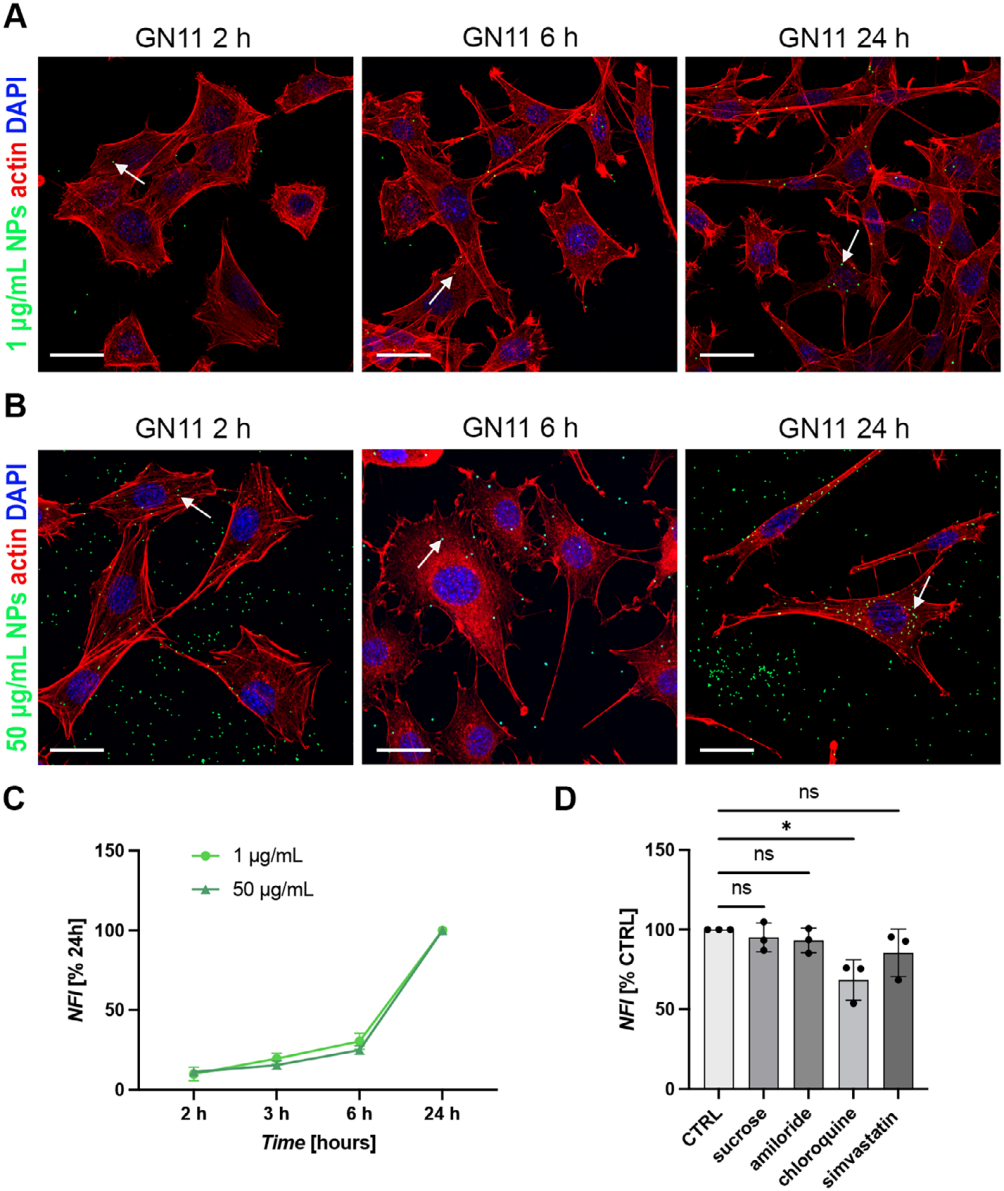
Non‐toxic concentrations of PS‐NPs are internalized into GN11 cells by clathrin and caveolin‐independent endocytosis. (A‐B) Confocal microscopy images of GN11 cells treated with 1 µg/mL (A) and 50 µg/mL (B) PS‐NPs for 2, 6, and 24 h. PS‐NPs (green) are internalized and localize in the cytoplasm (arrows). Nuclei are stained with DAPI (blue) and the actin cytoskeleton with phalloidin (red). Scale bars: 50 µm. (C) Flow cytometry analysis of PS‐NPs uptake inside GN11 cells treated with 1 (light green) and 50 µg/mL (dark green) concentrations for increasing durations (2, 3, 6, and 24 h). Data are shown as normalized fluorescence intensity (NFI, % of 24 h) as a function of PS‐NPs exposure time (*N* = 3). (D) NFI (% of untreated CTRL) in GN11 cells exposed to 50 µg/mL PS‐NPs for 24 h following 1 h pre‐treatment with endocytosis inhibitors. GN11 cells exposed to PS‐NPs but not pre‐treated with inhibitors were used as CTRL (*N* = 3; One Way ANOVA followed by Dunnett's multiple comparisons test, ^*^ *p* < 0.05, ns non significant; sucrose *p* = 0.9707, amiloride *p* = 0.9006, chloroquine *p* = 0.0163, simvastatin *p* = 0.3889).

Next, the cellular uptake dynamics of PS‐NPs in developing GnRH neurons were evaluated. GN11 cells were treated with non‐toxic concentrations of 1 and 50 µg/mL PS‐NPs for 2, 6, and 24 h, and intracellular localization of fluorescent PS‐NPs was qualitatively assessed by direct fluorescence imaging using confocal microscopy. As shown in Figure 2A,B, we detected particle internalization for both concentrations within 2 h of exposure. This internalization remained prominent even at later time points, including 6 and 24 h.

To quantify PS‐NP uptake, direct fluorescence of PS‐NP‐treated GN11 cells was measured by flow cytometry, as previously done for GT1‐7 cells. GN11 cells were exposed to 1 and 50 µg/mL PS‐NPs for varying times (2, 3, 6, and 24 h) before analysis. NFI as a function of exposure time for GN11 cells treated with 1 and 50 µg/mL PS‐NPs is shown in Figure 2C. Consistent with previous results, NPs were internalized by GN11 cells, with fluorescence intensity increasing over time up to 24 h following exposure to both concentrations. These results indicate a time‐dependent cellular uptake of PS‐NPs by immature GnRH neurons.

To identify the intracellular pathways involved in PS‐NPs internalization, GN11 cells were pre‐treated for 1 h with specific endocytic pathway inhibitors before exposure to 50 µg/mL PS‐NPs. PS‐NP uptake was then quantified by flow cytometry after 24 h. The same inhibitors used for GT1‐7 cells were chosen. As shown in Figure 2D, NFI was significantly reduced in GN11 cells pre‐treated with chloroquine, but not with the other inhibitors, as compared to control cells (mean % to CTRL ± SD: sucrose, 95.07 ± 9.05; amiloride, 93.15 ± 7.77; chloroquine, 68.37 ± 12.73; simvastatin, 85.45 ± 14.88). These findings suggest that immature immortalized GnRH neurons preferentially internalize PS‐NPs via generic, clathrin‐ and caveolin‐ independent, endocytosis, similar to mature GT1‐7 cells.

### 2.3 Exposure of GN11 Neuronal Cells to Non‐Toxic Concentrations of PS‐NPs Affects Cellular Migration via Actin Cytoskeleton Remodeling

Once established PS‐NP internalization by GN11 cells, we next assessed their biological effects by analyzing key cellular behaviors.

Increased production of reactive oxygen species (ROS) is one of the earliest responses triggered by micro‐ and nanoplastics exposure, both in vitro and in vivo [27, 28, 29, 30]. We therefore determined whether PS‐NPs elicit a similar ROS response in our cell model of immature GnRH neurons. GN11 cells were treated with PS‐NPs at concentrations of 1 and 50 µg/mL for 3 and 24 h, followed by assessment of ROS production by flow cytometry. We detected a significant increase in ROS levels after 24 h of treatment with both concentrations compared to untreated control cells, whereas the 3 h exposure did not produce significant changes (Figure S4A). These findings demonstrate that PS‐NPs induce a time‐dependent elevation of intracellular ROS in GN11 cells. Importantly, we confirmed that this generic stress response, triggered by selected non‐toxic PS‐NP doses in GN11 cells, was not accompanied by increased cell mortality (Figure S4B).

**FIGURE 3:**
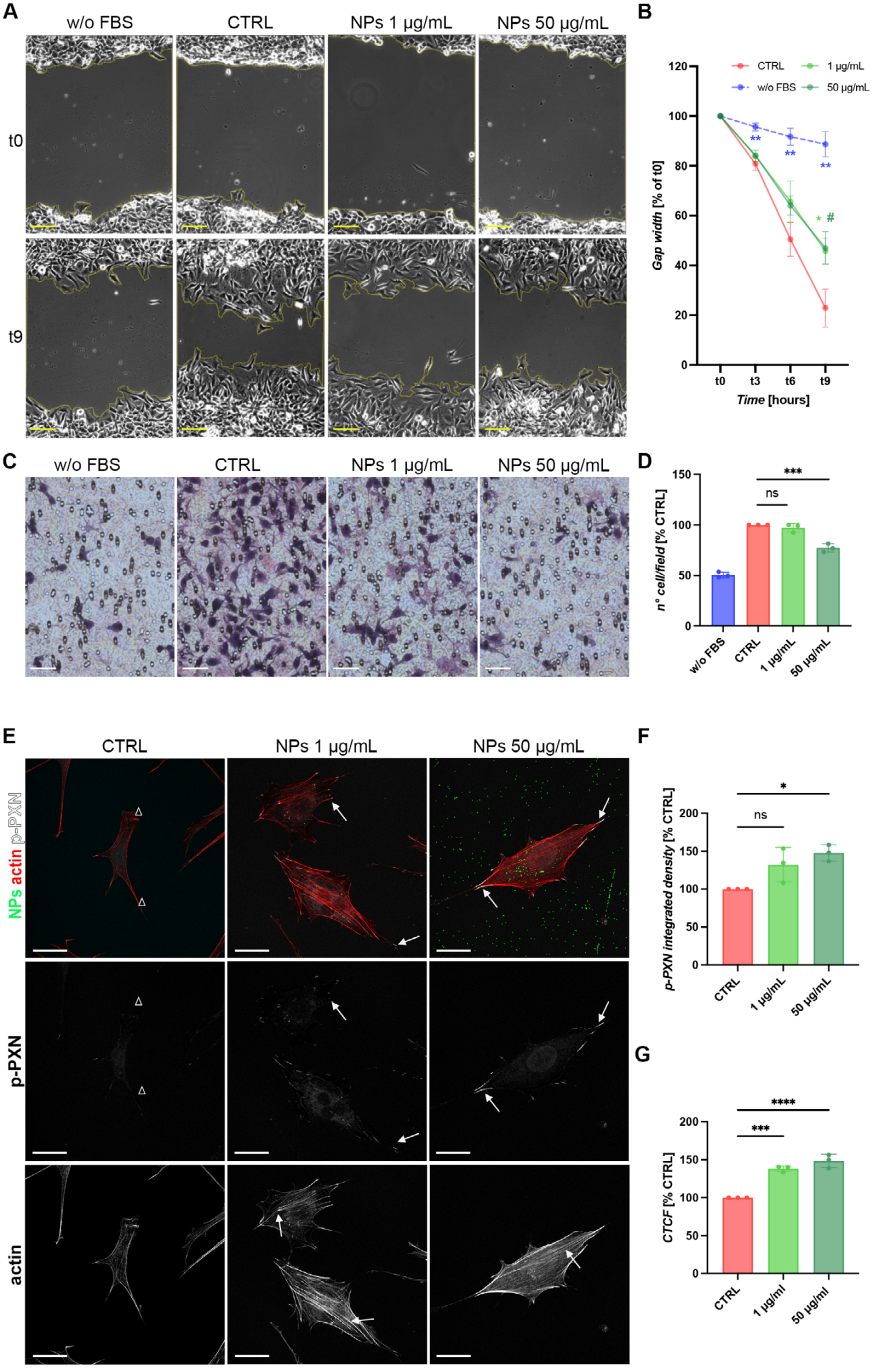
PS‐NPs impact GN11 neuron migration. (A‐B) Evaluation of migratory capacity of untreated GN11 cells (CTRL) and GN11 cells treated for 24 h with 1 and 50 µg/mL PS‐NPs toward FBS, assessed by scratch assays. Untreated GN11 cells exposed to DMEM without FBS were used as negative control (w/o FBS). (A) Representative phase‐contrast images showing scratch area reduction at scratching time (t0) and after 9 h (t9). Wound edges are highlighted in yellow. Scale bar: 500 µm. (B) Graph of gap width (% of t0) as a function of time (hours) after wound generation. Significant reduction in migratory capacity became evident 9 h after scratching for 1 (asterisks) and 50 µg/mL (hashtags) treated cells with respect to CTRL (*N* = 3; 2‐way ANOVA followed by Dunnet's multiple comparison test, ^**^ *p* < 0.01; ^*^ or # *p* < 0.05; t3: w/o FBS *p* = 0.0066, 1 µg/mL *p* = 0.3776, 50 µg/mL *p* = 0.3092; t6: w/o FBS *p* = 0.0059, 1 µg/mL *p* = 0.1520, 50 µg/mL *p* = 0.1106; t9: w/o FBS *p* = 0.0011, 1 µg/mL *p* = 0.0367, 50 µg/mL *p* = 0.0332). (C‐D) Assessment of chemomigratory capacity of untreated GN11 cells (CTRL) and GN11 cells treated for 24 h with 1 µg/mL and 50 µg/mL PS‐NPs toward complete DMEM (with FBS) by transwell assay. Migration of untreated cells to DMEM without FBS (w/o FBS) was used as negative control. (C) Representative light microscopy images showing migrated treated and untreated GN11 cells. Scale bar: 250 µm. (D) Graph of the number of migrated cells/field (% of CTRL), showing a significant reduction in cell migration for 50 µg/mL PS‐NP‐treated cells (*N* = 3; One Way ANOVA followed by Dunnett's multiple comparisons test, ***p < 0.001, ns non significant; 1 µg/mL *p* = 0.5376, 50 µg/mL *p* = 0.0004). (E) p‐PXN immunofluorescence staining of GN11 cells treated with 1 µg/mL and 50 µg/mL PS‐NPs (green) for 24 h. Untreated GN11cells were used as CTRL. Representative confocal microscopy images show increased p‐PXN (white) signal at the cell membrane of GN11 cells following PS‐NP exposure. Nuclei are stained with DAPI (blue) and the actin cytoskeleton with phalloidin (red). Single channels for p‐PXN and actin are also shown. Scale bars: 50 µm. (F) Quantification of p‐PXN fluorescent signal intensity by integrated density analysis (*N* = 3; One Way ANOVA followed by Dunnett's multiple comparisons test, ^*^ *p* < 0.05, ns non significant; 1 µg/mL *p* = 0.0607, 50 µg/mL *p* = 0.0124). (G) Quantification of F‐actin fluorescent signal intensity by CTCF analysis (*N* = 3; One Way ANOVA followed by Dunnett's multiple comparisons test, ^***^ *p* < 0.001, ^****^ *p* < 0.0001; 1 µg/mL *p* = 0.0003, 50 µg/mL *p* < 0.0001).

Next, to investigate the potential effects of PS‐NPs on GnRH neuronal migration and thereby their possible role in GD, GN11 cells were exposed to 1 and 50 µg/mL non‐toxic concentrations of PS‐NPs for 24 h, and two different migration assays were performed to assess both collective two‐dimensional migration and chemotactic migration through a porous membrane. First, scratch assays were used to track the migration of untreated (CTRL) and PS‐NP‐treated GN11 over time by measuring wound area reduction from 0 to 9 h after scratching. As shown in Figure 3A, bright‐field microscopy images revealed that GN11 cells exposed to PS‐NPs exhibited larger cell‐free areas after 9 h compared to CTRL, the latter having nearly covered the scratched region by this time point. Cell migration was significantly reduced in PS‐NP‐exposed cells treated with both 1 and 50 µg/mL PS‐NPs concentrations (Figure 3B, mean % to t0 ± SD: t3: CTRL 80.90 ± 2.69, w/o FBS 95.67 ± 1.53, 1 µg/mL 84.07 ± 2.18, 50 µg/mL 84.13 ± 1.10; t6: CTRL 50.47 ± 6.80, w/o FBS 91.73 ± 3.41, 1 µg/mL 65.67 ± 8.22, 50 µg/mL 64.10 ± 3.82; t9: CTRL 22.87 ± 7.64, w/o FBS 88.73 ± 5.14, 1 µg/mL 45.77 ± 5.15, 50 µg/mL 47.03 ± 6.53). These findings suggest that 500 nm PS‐NPs impair GN11 cell migration, with no concentration‐dependent reduction observed.

To further explore the migratory capacity of PS‐NP‐treated GN11 cells, transwell chemotactic assays were performed. As shown in Figure 3C,D the number of GN11 migrated cells after 3 h was significantly reduced when treated with 50 µg/mL PS‐NPs, while no effect was observed for cells treated with 1 µg/mL PS‐NPs (mean % to CTRL ± SD: 1 µg/mL, 97.20 ± 4.26; 50 µg/mL, 77.31 ± 4.13).

Overall, these assays suggest that, while collective migration of immature GnRH neurons in a two‐dimensional plane is affected by both 1 and 50 µg/mL PS‐NPs, chemotaxis is significantly reduced only at the higher concentration of 50 µg/mL PS‐NPs.

Given the intimate interplay between cell migration and adhesion, particularly through focal adhesion complexes, we next assessed whether PS‐NP exposure alters focal adhesion dynamics. To this end, GN11 cells were immunostained for phosphorylated paxillin (p‐PXN), a marker of active focal adhesions that links integrins to the actin cytoskeleton [31, 32]. As shown in Figure 3E, cells treated for 24 h with 1 or 50 µg/mL PS‐NPs exhibited increased p‐PXN accumulation at the cell membrane compared with control cells, indicating enhanced focal adhesion formation. Quantitative analysis confirmed a significant increase in p‐PXN signal intensity in PS‐NPs‐treated cells (Figure 3F; mean % to CTRL ± SD: 1 µg/mL, 132.16 ± 22.75 µg/mL; 50 µg/mL, 147.63 ± 10.72). Consistently, phalloidin staining revealed more organized actin filaments in PS‐NPs exposed cells compared to control cells, with a significant increase in actin fluorescence intensity, measured as corrected total cell fluorescence (CTCF) (Figure 3E,G; mean % to CTRL ± SD: 1 µg/mL, 137.94 ± 3.84 µg/mL; 50 µg/mL, 148.48 ± 8.85).

These data indicate that PS‐NPs promote focal adhesion assembly and actin cytoskeletal stabilization, consistent with the observed reduced cellular motility.

To assess if particle size might be critical to induce changes in GnRH neuron migration, we performed similar experiments using smaller diameter particles (50 nm). After verifying the absence of cytotoxicity at both previously tested concentrations (1 and 50 µg/mL), as well as their internalization into GN11 cells using the same methods as for the 500 nm particles, we found that smaller PS‐NPs did not influence cell migration under the same exposure conditions (Figure S5). This suggests that plastic particle size, rather than concentration, may play a more critical role in modulating the migratory capacity of GN11 cells.

### 2.4 Transcriptomic Profiling Reveals that PS‐NP Exposure Alters Key Regulators of GnRH Neuron Migration and Pubertal Onset

**FIGURE 4:**
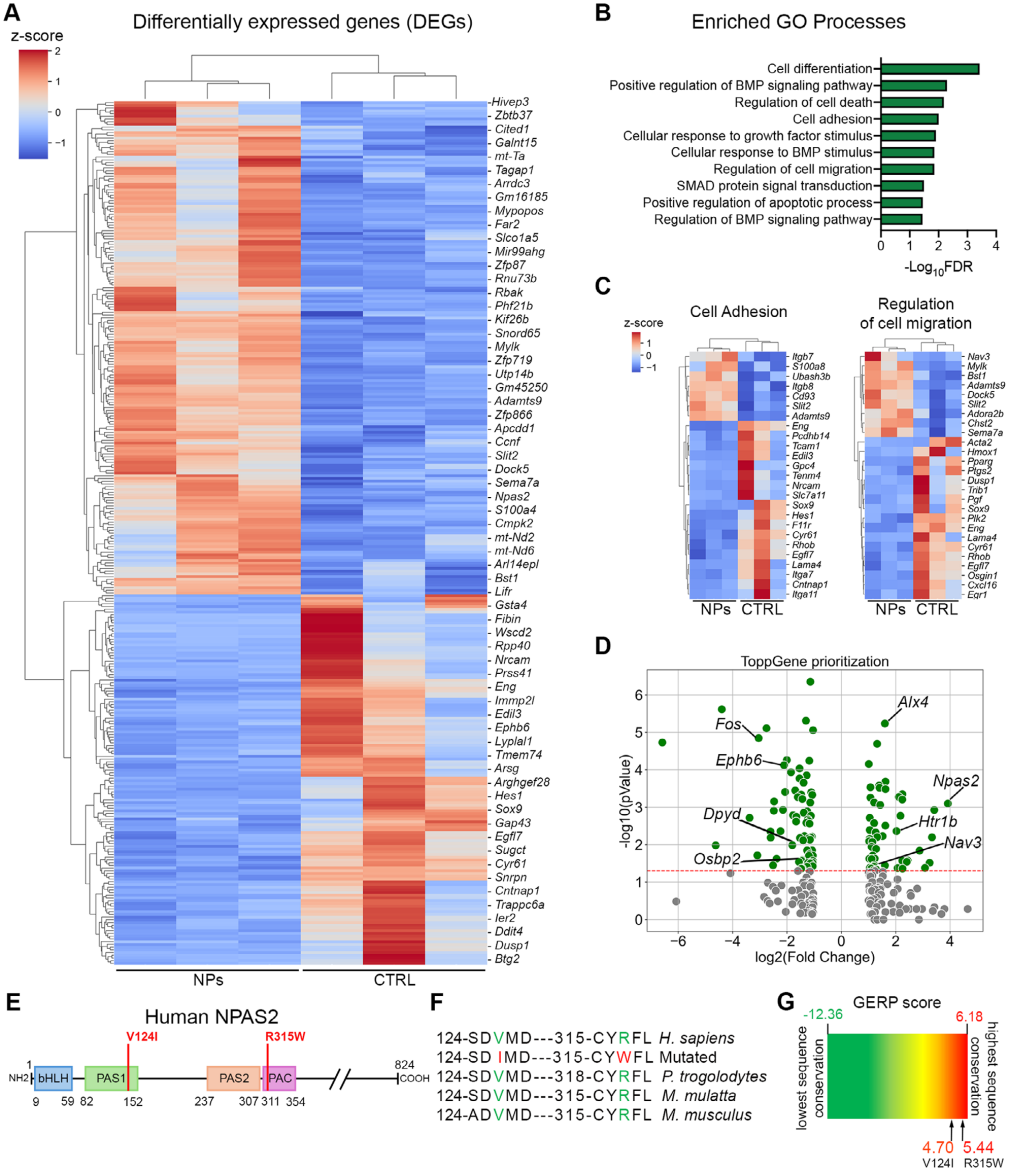
PS‐NPs impact GN11 transcriptomic signatures altering key regulators of GnRH neuron migration and pubertal onset. (A) Z‐scored gene expression values were clustered hierarchically (Euclidean distance metric) for DEGs between untreated (CTRL) and 50 µg/mL 500 nm PS‐NP‐treated GN11 cells (|log_2_FC| > 1, adjusted *p*‐value < 0.05). (B) Graph showing functionally enriched pathways computed on DEGs (FDR < 0.05). (C) Z‐ scored gene expression values for genes belonging to selected enriched pathways are shown. Heat‐maps representing examples of color‐coded expression levels of genes belonging to significantly enriched GO Processes. (D) Volcano plot of expression log_2_FC and ‐log_10_(*p*‐value) computed upon ToppGene prioritization. Differentially expressed genes (|log_2_FC| > 1) with *p*‐value < 0.05 are colored in green; all other genes are in gray. (E) Schematic of human NPAS2 protein with functional domains: bHLH (basic helix‐loop‐helix), PAS1 (Period‐Arnt‐Single minded 1), PAS2 (Period‐Arnt‐Single minded 2), and PAC (PAS‐associated c‐terminal). Positions of protein domains and of mutated amino acid residues within the protein sequence are indicated. (F) Alignment of partial protein sequences of indicated vertebrate NPAS2 orthologues showing that p.V124I and p.R315W residues are evolutionarily conserved in most species (green). *Homo sapiens* mutated residue is labeled in red. (G) Genomic evolutionary rate profiling of sequence constraint for the mutated *NPAS2* residues using GERP++ analysis provided a RS score of 4.70 (p.V124I) and 5.44 (p.R315W), which indicates a high level of conservation across all mammalian species.

To investigate the molecular mechanisms through which PS‐NPs affect GnRH neuron migration, we performed bulk RNA‐sequencing (RNA‐seq) analyses on GN11 exposed to 500 nm PS‐NPs. To this end, GN11 cells were treated for 24 h with 50 µg/mL PS‐NPs, the concentration that elicited the strongest inhibitory effect in migration assays. RNA‐seq was then performed on GN11 untreated (CTRL) and treated (PS‐NPs) samples (*N* = 3 per experimental group; GSE295898). Sample clustering by principal component analysis (PCA) revealed that PS‐NP treatment mainly impacted on gene expression signatures (Figure S6A). Among the two conditions, we found 317 differentially expressed genes (DEGs; |log_2_fold change (FC)| > 1 and adjusted *p*‐value < 0.05) (Figure 4A); specifically, 181 genes were up‐regulated in GN11 treated with PS‐NPs, whilst 136 genes were down‐regulated (Figure S6B). Interestingly, among DEGs we found genes relevant for GnRH neuron development and biology (*Slit2*, *Lifr* and *Hes1*), HPG axis development and function (*Dusp1* and *Sox9*), as well as one gene recently identified as new candidate gene in central reproductive disorders with GD (*Gap43*) [33, 34, 35, 36, 37, 38, 39, 40, 41]. Notably, we also identified two genes (*Sema7a* and *Dusp6*) already implicated in the pathogenesis of developmental forms of GD (Figure 4A) [42, 43, 44, 45]. Functional enrichment analysis computed on DEGs and based on Gene Ontology (GO) Biological Processes annotations was performed using reString software and revealed significantly enriched pathways (false discovery rate, FDR < 0.05) related to cell differentiation, cell adhesion, and cell migration (Figure 4B,C), in agreement with our in vitro results showing impaired adhesion and migration of PS‐NP‐treated GN11 cells.

To identify, among DEGs, those that could be potentially implicated in GD pathogenesis, ToppGene software was applied to rank candidates based on functional or structural similarity with a list of genes involved in GnRH neuron developmental processes with identified patient variants or in vivo evidence (Table S1) [46, 47, 48]. Following DEG prioritization, we cross‐referenced the top‐ranked genes (*p‐*value < 0.05) with large GWAS datasets on pubertal timing, and we revealed several common genes, including *HTR1B*, *NAV3*, *EPHB6*, *FOS*, *OSBP2*, *DPYD*, and *ALX4* (Figure 4D) [49, 50, 51, 52]. Finally, we matched prioritized DEGs with exome sequencing from a cohort of > 400 probands with GD and a second cohort of 77 probands with constitutional delayed puberty, a non‐permanent form of GD, to identify possible novel candidate genes of these disorders [53]. We identified two heterozygous missense variants (ENST00000335681.5: c.943C>T, p.R315W and c.370G>A, p.V124I) in Neuronal PAS domain protein 2 (*NPAS2)* gene (Figure 4E), encoding for a transcription factor involved in circadian rhythm regulation [54].

The male proband carrying the *NPAS2* p.R315W variant presented aged 17.1 years with delayed pubertal progression and lack of pubertal growth spurt. He reported having had late onset of puberty and had achieved testes volume of 5–6 mL by the age of first review. He was noted to have mild gynecomastia bilaterally but no micropenis, synkinesia or other clinical features associated with GD. He was otherwise healthy with a normal sense of smell (reported) and no previous illnesses. He had achieved appropriate educational attainment for his age. His initial investigations showed a delayed bone age of 13.5 years at a chronological age of 17.1 years, with biochemical evidence of GD (LH 0.8 IU/L, FSH 2.2 IU/L, testosterone 1.0 nmol/L). Inhibin B concentrations, thyroid function tests, cortisol, and IGF1 were in the normal range for age (Table S2). MRI scan showed a normal appearance of the pituitary gland, stalk, and hypothalamus. There was no family history of GD. He was monitored without treatment, and testes volumes increased spontaneously, to 15–20 mL by the age of 18.3 years, consistent with severe delayed puberty. Pathogenicity analyses showed that the missense p.R315W variant is conserved across species (Figure 4F,G) and predicted as pathogenic moderate according to bioinformatic tools and ACMG guidelines (Table 1).

**TABLE 1:** Chromosome position on human GRCh38 genome assembly, nucleotide substitution, amino acid substitution, and bioinformatic predictions of the identified *NPAS2* missense variants.

Chr^a^	Position	nt^b^ sub^c^	aa^d^ sub	PopMax AF^e^	CADD	REVEL	AlphaMissense
2	100968316	c.943C>T	p.(R315W)	6.197 × 10^−7^	31	Uncertain significance	Likely pathogenic
2	100948241	c.370G>A	p.(V124I)	0	21	Benign (moderate)	Benign (moderate)

^a^ Chromosome

^b^ Nucleotide

^c^ Substitution

^d^ Amino acid

^e^ Maximum population allele frequency.

The male proband carrying the *NPAS2* p.V124I variant presented aged 14.5 years with pubertal delay and short stature (height SDS ‐2.8). His initial investigations showed a delayed bone age of 12.5 years at a chronological age of 14.5 years. He was further advanced through puberty than the first proband described above, having achieved testes volumes of 6 mL by the age of 14.8 years with LH 3.1 IU/L, FSH 7.3 IU/L, and testosterone 9.7 nmol/L. However, he had a late pubertal growth spurt with height velocity of 9.54 cm/year at the age of 15.3 years in keeping with delayed puberty (Table S3). He was otherwise healthy with appropriate educational achievement for his age. His mother also reported a history of delayed puberty with menarche at age 14 years. Sequencing data was not available from family members. The p.V124I variant, not seen in population databases of healthy individuals, is also well conserved across species (GERP score 4.7) and predicted as a variant of unknown significance (Table 1).

## 3 Discussion and Conclusion

This study investigates the hitherto unexplored effects of PS‐NPs on immature (GN11) and mature (GT1‐7) GnRH in vitro neuronal models, providing novel evidence that environmental contaminants may impact reproductive functions via direct activities on the neuroendocrine system.

The use of GN11 and GT1‐7 murine cell lines overcomes the challenge of isolating primary GnRH neurons, a sparse population of neurons that develop in a short time‐window [55]. These models are well‐characterized tools for studying GnRH neuron physiology as well as to conduct toxicological screenings [56, 57, 58, 59, 60, 61].

Our findings demonstrate that 500 nm PS‐NPs are readily internalized by both GN11 and GT1‐7 cells through a clathrin‐ and caveolin‐independent endocytic route. This non‐canonical uptake is consistent with reports that larger NPs enter cells via generic endocytosis when their size exceeds the optimal range for clathrin or caveolae‐mediated internalization [23]. Importantly, internalization occurred at non‐toxic concentrations, indicating that observed defects reflect functional dysregulation rather than cytotoxicity.

In GT1‐7 cells, PS‐NP exposure impaired *Gnrh1* transcription and reduced intracellular GnRH peptide levels, ultimately resulting in a decrease in GnRH hormone secretion. These findings aligns with in vivo evidence of NPs ability to cross the BBB and reduce hypothalamic GnRH release [17, 18, 62]. Given the central role of GnRH in orchestrating puberty and fertility, even modest reductions in its synthesis may have substantial physiological consequences [9].

In GN11 cells, PS‐NPs affected processes associated with early GnRH neuron development. Exposure induced a time‐dependent increase in ROS, an early and common response to NP exposure [27, 63, 64]. Although ROS did not compromise vitality at the concentrations tested, they likely acted as a stress signal capable of reshaping cytoskeletal dynamics [65, 66, 67]. Consistent with this, 500 nm PS‐NPs significantly impaired GN11 migration in both wound‐healing and transwell assays. Collective two‐dimensional migration was impaired at both concentrations (1 and 50 µg/mL), whereas chemotactic migration was affected only at 50 µg/mL. This divergence can be explained by fundamental differences between the assays: wound healing captures the collective two‐dimensional migration across a wound gap, primarily assessing the ability of cells to migrate as a monolayer, whereas transwell assay requires individual chemotactic motility through a porous membrane in response to a directional stimulus. Thus, those assays provide complementary insights into different aspects of cell motility.

Mechanistically, PS‐NPs promoted focal adhesion assembly, evidenced by increased phosphorylated paxillin and more organized actin filaments. Stabilized adhesions are known to hinder the turnover required for forward movement, consistent with previous GN11 studies showing that enhanced adhesion restricts motility [26]. These results identify altered focal adhesion dynamics as a kay mechanism through which PS‐NPs impair migration in developing GnRH neurons.

Interestingly, migration defects were strongly size‐dependent. Smaller 50 nm PS‐NPs, despite efficient uptake, did not impair GN11 cell motility. While smaller NPs are often considered more harmful due to their surface‐area‐to‐volume ratio, multiple studies show that larger PS‐NPs can induce greater ROS production and more pronounced cytoskeletal alterations [68, 69, 70, 71]. Our data support a model in which particle size influences the magnitude of downstream cellular responses, with 500 nm PS‐NPs more effectively perturbing motility‐related pathways in GN11 cells than their 50 nm counterparts.

Transcriptomic profiling of PS‐NPs exposed GN11 cells further revealed differential expression of genes regulating cell adhesion, cytoskeletal organization, and migration, mirroring the observed phenotypes. The mixed pattern of up‐ and down‐regulated genes reflects the complexity of cytoskeletal and adhesive remodeling triggered by PS‐NP exposure, with multiple pathways adjusting in opposite directions to influence overall migratory behavior. Upregulated genes include anti‐migratory regulators such as *Slit2*, a repulsive axon guidance cue involved in GnRH neuron migration, *Adamts9*, which suppresses cell motility through Akt/p53 signaling, as well as integrins such as *Itgb7* and *Itgb8*, which strengthen cell‐matrix adhesion [72, 73, 74, 75]. Conversely, several pro‐migratory genes were downregulated: Rho GTPases like *Rhob*, a driver of lamellipodia formation and focal adhesion turnover, and *Sox9*, a regulator of progenitor cell migration [76, 77, 78]. Together, these shifts create a transcriptional environment favoring stronger adhesion and reduced motility.

Several DEGs were also implicated in GnRH neuronal development and HPG axis regulation, further suggesting that PS‐NPs perturb molecular networks essential to reproductive maturation [34, 35, 36, 37, 38, 39, 40, 41, 42, 43, 44, 45].

Integration of prioritized DEGs with a large‐scale GWAS dataset on age at menarche identified shared candidate genes such as *HTR1B*, *NAV3*, *EPHB6*, *FOS*, *OSBP2* [49]. Notably, *HTR1B*, *OSBP2* and *DPYD* were independently identified in other GWAS analyses, reinforcing their potential role in the genetic regulation of pubertal timing [50]. Among the top‐ranked candidates, *ALX4* emerges as particularly compelling, having been linked to hypogonadism through a homozygous nonsense mutation and associated with male puberty timing in multi‐trait GWAS [51, 52]. These converging lines of evidence suggest *ALX4* as a compelling NP‐responsive gene relevant to GD.

*NPAS2* also emerged as an environmentally responsive gene mis‐regulated by PS‐NPs and harboring rare variants in individuals with GD or delayed puberty. NPAS2 is a basic helix‐loop‐helix‐PAS (bHLH‐PAS) transcription factor that functions as a core component of the molecular circadian clock to regulate rhythmic gene expression [54]. It is highly expressed in the mouse adult brain, including the suprachiasmatic nucleus and cerebral cortex, and has overlapping functions with CLOCK, another circadian regulator [79, 80]. *Npas2* knockout mice exhibit disrupted circadian rhythms and altered behavioral and metabolic phenotypes, supporting its role in neuroendocrine regulation [81, 82, 83]. Intriguingly, other NPAS family members, such as NPAS3 and NPAS4, have been implicated in neurodevelopment and psychiatric disorders, suggesting a broader involvement of this protein family in brain function and plasticity [84, 85, 86]. Altogether, these evidences hint at *NPAS2* potential role as an environmentally responsive gene involved in reproductive and neurodevelopmental disorders.

Beyond direct effects on GnRH neurons, our findings contribute to broader concerns regarding PS‐NPs as pervasive environmental contaminants. As plastic waste accumulates globally, it degrades into nanoscale plastic debris that infiltrate ecosystems and food chains [87, 88, 89]. These particles can enter the human body through ingestion, inhalation, or dermal contact, and have been shown to cross biological barriers, including the placenta, raising concerns about fetal exposure during critical windows of neurodevelopment [90, 91, 92]. NPs may exert toxic effects not only through direct cellular interactions but also by acting as carriers for endocrine‐disrupting chemicals, amplifying their biological impact [93, 94, 95, 96]. Such interactions may disrupt hormonal signaling pathways and impair gametogenesis, potentially contributing to declining fertility worldwide [12, 13, 97, 98, 99, 100].

Collectively, our findings reveal a dual vulnerability of developing and mature GnRH neurons to 500 nm PS‐NPs, impairing both neuronal migration and GnRH hormone production. By interfering with those processes, essential for reproductive axis development, PS‐NPs may contribute to the etiology of reproductive disorders such as GD, a condition still only partially explained by genetic variants [11]. Notably, the observed cellular defects parallel transcriptomic changes in genes associated with GnRH neuron development and reproductive disorders, highlighting PS‐NPs as potential environmental disruptors of neuroendocrine regulation. The interplay or synergy between PS‐NP exposure and individual genetic susceptibility likely reflects the multifactorial nature of conditions such as GD. Further research is needed to clarify and characterize these interactions, including validating the effects of PS‐NPs on the GnRH system in vivo using relevant animal models. Such studies will be essential to assess the broader health risks associated with NP exposure and to guide strategies aimed at mitigating their impact. A deeper understanding of how PS‐NPs modulate reproductive pathways may ultimately improve the diagnosis and treatment of idiopathic infertility linked to environmental exposures.

## 4 Experimental Section/Methods

### 4.1 Polystyrene Nanoplastics (PS‐NPs)

PS‐NPs used for the experiments of this study have a diameter of 50 nm (Thermo Fisher Scientific, Milan, Italy; cat. G50) or 500 nm (Thermo Fisher Scientific, Milan, Italy; cat. G500) and contain a green, fluorescent dye embedded within the polymer matrix. They are packaged in deionized water with traces of surfactant and preservative to inhibit aggregation and promote stability. These PS‐NPs are provided as aqueous suspensions with 1% solids by weight, a refractive index of 1.59, and a density of 1.06 g/cm^3^. For intracellular ROS quantification only, experiments were performed using 500 nm PS‐NPs incorporating a matrix‐embedded red fluorescent dye (Thermo Fisher Scientific, Milan, Italy; cat. R500) instead of the green‐labelled PS‐NPs. The use of red‐labelled PS‐NPs prevented fluorescence overlap with the green reagent employed for intracellular ROS detection. These red PS‐NPs displayed the same physicochemical properties as the green‐labelled counterparts used in the other experiments.

### 4.2 Cell Lines

GT1‐7 and GN11 cells were maintained at 37°C in a humidified 5% CO_2_ incubator in DMEM (Euroclone, Milan, Italy; cat. ECB7501L) supplemented with 10% (v/v) heat‐inactivated fetal bovine serum (FBS; Life Technologies, Monza, Italy; cat. 10270106), 2 mM L‐Glutamine (Euroclone, Milan, Italy; cat. ECB3000), 100 IU/mL penicillin and 100 µg/mL streptomycin (Euroclone, Milan, Italy; cat. ECB3001D), referred to complete DMEM medium. Sub‐confluent cells were harvested by trypsinization and cultured in 57 cm^2^ dishes at a density of 8 × 10^4^ (GN11) and 5 × 10^5^ cells (GT1‐7) cells/dish for routine passaging.

### 4.3 Cell Viability Assays

GT1‐7 and GN11 cell vitality were assessed by monitoring the conversion of MTT [3‐(4,5‐dimethylthiazol‐2‐yl)2,5‐diphenyltetrazolium bromide] to formazan. Briefly, GT1‐7 and GN11 cells were seeded in 24‐well plates at a density of 8 × 10^4^ and 3 × 10^4^ cells/well, respectively, and allowed to adhere for 24 h (h). Cells were then treated with 50/500 nm PS‐NPs in complete medium at the following concentrations: 50, 100, 250, 500 ng/mL, 1, 12.5, 25, 50, 100, and 200 µg/mL. Untreated cells grown in complete medium were used as positive controls, while untreated cells grown in DMEM without FBS were used as negative controls. Cell vitality was assessed 24 and 48 h after PS‐NPs treatment by adding the MTT solution (450 µg/mL in serum‐free white medium, Merck, Darmstadt, Germany; cat. M5655) to each well and incubating for 30 min (min) at 37°C. After the incubation, the MTT solution was removed, isopropanol (Merck, Darmstadt, Germany; cat. 59300) was added to each well, and the multi‐well was shaken for 10 min at room temperature (RT). The *A*_550_ of plates was measured using the EnSpireTM Multimode Plate Reader (PerkinElmer, Shelton, CT, USA). Three replicates of each treatment were assayed. The viability of cells was calculated by plotting the absorbance (% of positive CTRL) of treated and control cells at each time point (24 and 48 h).

### 4.4 Direct Fluorescent Imaging

Confocal microscopy images were acquired after cell fixation and cytoskeleton and nuclei staining to qualitatively assess particle internalization in GT1‐7 and GN11 cells. GT1‐7 and GN11 cells were seeded on 13‐mm circular coverslips in 24‐well plates at the density of 5 × 10^4^ and 2.5 × 10^4^ cells/well, respectively. The day after plating, cells were treated with 1 and 50 µg/mL suspensions of 50/500 nm PS‐NPs in complete DMEM for 24, 6 and 2 h and fixed with 4% paraformaldehyde (PFA; Life Technologies, Monza, Italy; cat. 28908) for 15 min at RT. Nuclei were counterstained DAPI (4′,6‐diamidin‐2‐phenylindole, 1:10,000; Cell Signaling, Leiden, Netherlands; cat. 4083). To detect F‐actin filaments, cells were stained with TRITC‐conjugated phalloidin (1:400; Merck, Darmstadt, Germany; cat. P1951) for 30 min at 37°C. Confocal images were acquired with a Zeiss LSM900 laser scanning confocal microscope and a Zeiss Plan‐Apochromat 40× objective (NA 1.3). ZEN 3.0 software (v.3.0.79.0006; Zeiss, Oberkochen, Germany) was used to process z‐stacks at 0.25 µm intervals and generate maximum intensity projection images. Adobe Photoshop 2023 software (Adobe, San Jose, CA, USA) was used to prepare the presented images.

### 4.5 Flow Cytometer Uptake Assay

Particle internalization in GT1‐7 cells was evaluated using flow cytometry following NPs exposure for varying exposure times. Cells were seeded in 6‐well plates at a density of 2 × 10^5^ cells/well and incubated at 37° with 5% CO_2_. After 24 h, the medium was removed, and cells were washed once with Phosphate Buffered Saline (PBS; Euroclone, Milan, Italy; cat. ECB4004L) before incubation with 1 and 50 µg/mL 500 nm PS‐NPs in fresh complete medium for 2, 3, 6 and 24 h at 37° with 5% CO_2_. Following incubation, cells were harvested, centrifuged twice at 1,200 rpm for 5 min (after resuspension in 1 mL PBS), and finally resuspended in 200 µL of PBS. Samples were analysed with BD FACS Celesta flow cytometer with BD FACS Diva software (BD Biosciences, Franklin Lakes, NJ, USA) to calculate the percentage of positive events and their mean fluorescence intensity. Unexposed cells served as controls for basal fluorescence levels. Three replicates of each treatment were performed.

Particle internalization in GN11 cells was evaluated using flow cytometry after treatment with 500 nm PS‐NPs for varying exposure times. Cells were seeded in 12‐well plates at the density of 5 × 10^4^ cells/well. The day after plating, cells were treated with 1 and 50 µg/mL PS‐NPs in complete medium for 2, 3, 6, and 24 h. Three replicates of each treatment were assayed. Untreated GN11 cells were used to normalize for cell auto‐fluorescence. Following PS‐NPs treatment, GN11 cells were harvested, pelleted upon centrifugation at 12,000 rpm for 5 min at RT, wash in PBS, pelleted again, and mechanically disaggregated by pipetting to reach a single cell suspension in 100 µL PBS for flow cytometry analysis. Flow cytometry assays were performed through the Novocyte3000 instrument (Agilent, Palo Alto, CA, USA). Data were analyzed with Novoexpress software (v.1.4.1; Agilent, Palo Alto, CA, USA). The following parameters were used: mean FITC‐H as reading channel, stop condition 80 µL, flow rate 60 µL/min, core diameter 16 µm, threshold (FSC‐H) 100,000.

### 4.6 Endocytosis Inhibitors Treatment and Flow Cytometer Uptake Analysis

To study the specific internalization pathway exploited by PS‐NPs to enter GT1‐7 and GN11 cells, a pre‐treatment with different inhibitors of cellular uptake was conducted before PS‐NPs exposure. Briefly, GT1‐7 and GN11 cells were seeded in 12‐well plates at a density of 1.2 × 10^5^ and 5 × 10^4^ cells/well, respectively, and incubated at 37° with 5% CO_2_. Two days after plating, medium was removed, cells were washed once with PBS and then pre‐treated for 1 h at 37° with 45 mM sucrose (PanReac AppliChem, Darmstadt, Germany; cat. A2188), 10 µg/mL amiloride (Merck, Darmstadt, Germany; cat. A7410), 20 µM chloroquine (Merck, Darmstadt, Germany; cat. C6628) and 2.5 µM simvastatin (Merck, Darmstadt, Germany; cat. S6196) endocytosis inhibitors. After the treatment with the above‐mentioned inhibitors, the medium was removed, cells were washed once with PBS, and then exposed to 50 µg/mL 500 nm PS‐NPs in complete medium for 24 h at 37° with 5% CO_2_ prior to the assay using the flow cytometer. Three replicates of each pre‐treatment were assayed. GT1‐7 and GN11 cells treated with PS‐NPs but not pre‐treated with inhibitors of endocytic pathways were used as controls. Flow cytometry analysis was performed as above using the Novocyte3000 instrument (Agilent, Palo Alto, CA, USA) for GN11 cells and the Fortessa BD X20 (BD Biosciences, Franklin Lakes, NJ, USA) for GT1‐7 cells.

### 4.7 RNA Extraction and qPCR

Total RNA was isolated from GT1‐7 cells treated with 1 and 50 µg/mL PS‐NPs in complete medium for 24 h using TriFast II Reagent (Euroclone, Milan, Italy; cat. EMR517100). RNA (1 µg) was reverse transcribed with the High‐Capacity cDNA Reverse Transcription Kit (Thermo Fisher Scientific, Milan, Italy; cat. 4368814). Quantitative PCR (qPCR) was performed using Luna Universal qPCR Master Mix (NEB, Ipswich, MA, USA; cat. M3003X) on a CFX96 Real‐Time System (Bio‐Rad; Segrate, Italy), according to manufacturer indications and using specific oligonucleotides for murine *Gnrh1* (5’‐CGTTCACCCCTCAGGGATCT‐3’, 5’‐CTCTTCAATCAGACTTTCCAGAGC‐3’. Gene expression was normalized to *Gapdh* (5’‐ CATCCCAGAGCTGAACG ‐3’, 5’‐ CTGGTCCTCAGTGTAGCC ‐3’) using the ΔΔCt method.

### 4.8 Immunocytochemistry and Quantification

GnRH immunocytochemistry on GT1‐7 cells was performed as previously described for GN11 cells [101]. Briefly, rabbit anti‐GnRH primary polyclonal antibody was used (1:1000; Immunostar, Hudson, WI, USA; cat. 20075). After overnight incubation with primary antibody, cells were incubated with biotinylated goat anti‐rabbit antibody (1:400; Vector Laboratories, Newark, CA, USA; cat. BA‐1000) and then developed with the ABC kit (Vector Laboratories, Newark, CA, USA; cat. PK6100) and 3,3‐diaminobenzidine (Merck, Darmstadt, Germany; cat. D4418). Images were acquired with a Zeiss Axioskop 2 plus brightfield microscope and a Zeiss Plan‐NEOFLUAR 20× objective (NA 0.5). For each condition, three images per well were taken, and each condition was plated in triplicate. Quantification of GnRH staining was performed using ImageJ (v.1.54 g; NIH, Bethesda, MD, USA) software by measuring the integrated density of pixels in each image. P‐PXN immunocytochemistry on GN11 was performed as previously described [26]. Briefly, GN11 cells were grown on 13‐mm coverslips at the density of 1.5 × 10^4^ cells/well, incubated for 24 h with 1 and 50 µg/mL PS‐NPs in complete DMEM and fixed with 4% PFA for 10 min at RT. To detect F‐actin filaments, cells were stained with TRITC‐conjugated phalloidin as described above. To detect focal contacts, cells were immunolabelled with a rabbit anti‐phospho‐paxillin antibody (P‐PXN, 1:150; Cell Signaling, Leiden, Netherlands; cat. 69363) overnight at 4°C followed by an anti‐rabbit Alexa Fluor 647‐conjugated secondary antibody (1:200; Jackson ImmunoResearch, Cambridgeshire, United Kingdom; cat. 711‐606‐152). Confocal images were acquired with a Zeiss LSM900 laser scanning confocal microscope and a Zeiss Plan‐Apochromat 40× objective (NA 1.3). ZEN 3.0 software (v.3.0.79.0006; Zeiss, Oberkochen, Germany) was used to process z‐stacks at 0.25 µm intervals and generate maximum intensity projection images. Quantification of p‐PXN and F‐actin staining was performed using ImageJ (v.1.54 g; NIH) software by measuring the integrated density of pixels and corrected total cell fluorescence (CTCF), respectively, in each image. Adobe Photoshop 2023 software (Adobe, San Jose, CA, USA) was used to prepare the presented images.

### 4.9 NanoHPLC‐HRMS Analysis of GnRH

For the nanoHPLC‐HRMS analysis of GnRH peptide levels in the conditioned medium (CM) of GT1‐7 cells, we followed a previously published protocol with minor modifications [102]. Briefly, CM from untreated (CTRL) cells and from cells treated with 1 or 50 µg/mL PS‐NPs in complete DMEM for 24 h GT1‐7 cells were collected and centrifuged at 1,800 rcf × 5 min. The supernatant was then recovered. To obtain a measurable amount of GnRH, 2 mL of CM were subsequently treated with formic acid (VWR International, Milan, Italy, cat. 20318.297) in cold acetonitrile (VWR International, Milan, Italy, cat. 83640.320) and incubated at ‐20°C for 1 h for protein precipitation. The supernatant was collected after centrifugation (10,000 rpm × 10 min, RT; JOUAN SA, Milan, Italy) and freeze‐dried overnight using a CentriVap vacuum system (Labconco Co., Kansas City, MO, USA). The residue was reconstituted with the Loading Solution (LS) composed by 1 mL of 3% acetic acid (Merck, Milan, Italy; cat. 100063.1000) and 1% trifluoroacetic acid (Merck, Milan, Italy; cat. T6508) Milli Q water solution. After sonication and centrifugation, the reconstituted samples were purified with a Solid‐Phase Extraction (Strata‐X 33 µm Polymeric Reversed Phase, 60 mg/3 mL; Phenomenex, Bologna, Italy). The cartridges were equilibrated with methanol and LS, loaded with samples, washed with a mixture of LS/methanol (70:30 v/v), and finally eluted with methanol (VWR International, Milan, Italy; cat. 20837.320) and 3% acetic acid (70:30 v/v). The eluted solution was freeze‐dried using a CentriVap and reconstituted with 100 µL 0.1% formic acid in ultra‐pure water (ACS reagent, for ultratrace analysis, Merck, Milan, Italy; cat. 102696280) and injected into the nano‐HPLC‐HRMS system. All samples were prepared and analyzed in triplicate. Chromatographic separation and analysis were performed using a nano‐HPLC system (Dionex Ultimate 3000, Thermo Fisher Scientific, Milan, Italy) coupled with an Orbitrap Tribrid Fusion analyzer (Thermo Fisher Scientific, Milan, Italy). The chromatographic separation was lead with a Pep‐Map C18 column (2 µm, 100 Å, 75 µm × 15 cm; Thermo Fisher Scientific, Milan, Italy) preceded by a nano‐preconcentration column (C18 PepMap trap cartridge 100 Å, 5 µm, 0.3 mm × 5 mm; Thermo Fisher Scientific, Milan, Italy). Mobile phase was composed of 0.1% formic acid in ultra‐pure water (A) and 0.1% formic acid in acetonitrile/ultra‐pure water 8/2 (B) for the nano‐column, and trifluoroacetic acid 0.05% in ultra‐pure water/acetonitrile 98/2 (P) for the pre‐concentration column. The run gradient was set as follows: 5% of B was maintained for 8 min to pre‐concentrate the analyte, then it increased from 5% to 90% in 35 min. Then, the column went back to the initial conditions for a total run time of 45 min. Injection volume and flow rate were3 µL and 300 nL/min, respectively. After the nanoHPLC separation, the analyte was delivered to HRMS orbitrap analyzer through a nano‐ESI source. This latter worked with spray positive voltage at 2000 V and ion transfer tube temperature of 275°C. The HRMS detection was achieved acquiring a full mass event in the range between 100–1500 *m/z*. A dedicated MS/MS event using CID (Collision Induced Dissociation) activation mode at 27 V was set to obtain the fragmentation of double charged precursor ion of GnRH (*m/z* 591.7). The resolving power was set at 60K for all the scans. Identification and quantification of GnRH in medium sample was obtained using Xcalibur Software (Thermo Fisher Scientific, version 4.0.27.13) and an external calibration curve in the concentration range between 1 and 100 ng/mL. Limit of detection (LOD) and of Quantitation (LOQ) were determined using the formula of three‐ and ten‐times standard deviation of blank analysis (*n* = 10). The values of LOD and LOQ were 0.011 and 0.036, respectively.

### 4.10 Reactive Oxygen Species (ROS) Assay

Oxidative stress and intracellular ROS levels in GN11 cells were assessed using the CellROX Green Reagent (Thermo Fisher Scientific, Milan, Italy; cat. C10444). Fluorescence was measured in live cells by flow cytometry at 485/520 nm, following manufacturer's instructions. Briefly, GN11 cells were seeded in 24‐well plates at a density of 5 × 10^4^ cells/well and incubated in complete medium at 37° with 5% CO_2_. After 24 h, the medium was replaced with fresh complete medium containing 1 and 50 µg/mL red 500 nm PS‐NPs, and cells were incubated for 3 or 24 h at 37° with 5% CO_2_. Untreated cells were used as CTRL. As a positive control, GN11 cells were treated with tBHP 250 µM *tert*‐butyl hydroperoxide (tBHP; Merck, Darmstadt, Germany; cat. 458139‐25ML) in DMEM w/o FBS for 30 min at 37°C with 5% CO_2_. Following PS‐NPs or tBHP exposure, cells were washed once with PBS and incubated with 5 µM CellROX Green Reagent in PBS, shielded from light, for 30 min at 37° with 5% CO_2_. Stained cells were then harvested, centrifuged at 1,200 rpm for 5 min, and resuspended in 200 µL of PBS for flow cytometry analysis. Green fluorescence intensity was acquired using the Novocyte3000 instrument (Agilent, Palo Alto, CA, USA), and data were analyzed with Novoexpress software (v.1.4.1; Agilent, Palo Alto, CA, USA), using the same parameter settings described above.

### 4.11 DRAQ7 Cell Viability Test

Cell membrane integrity and mortality in GN11 cells were evaluated using the far‐red fluorescent viability dye DRAQ7 (Immunological Sciences, Rome, Italy; cat. DR50050), which selectively stains nuclei of dead or membrane‐compromised cells. GN11 cells were seeded in 12‐well plates at a density of 1.35 × 10^5^ cells/well and cultured in complete medium at 37° with 5% CO_2_. After 24 h, the medium was replaced with fresh complete medium containing green 500 nm PS‐NPs at concentrations of 1 and 50 µg/mL, and cells were incubated for 3 or 24 h under standard conditions. Untreated cells were used as CTRL. To assess whether intracellular ROS generation was associated with increased cell death, cells were exposed to 250 µM tBHP (Merck, Darmstadt, Germany; cat. 458139‐25ML) in DMEM w/o FBS. As a positive control for cytotoxicity, GN11 cells were treated with 200 µM hydrogen peroxide (H_2_0_2_) in DMEM w/o FBS for 30 min at 37°C. After treatments, the culture medium was collected, and adherent cells were harvested and combined with the corresponding medium. Cell suspensions were centrifuged at 1,200 rpm for 5 min, washed once with PBS, centrifuged again, and resuspended in DRAQ7 working solution (3 µM in PBS). After 10 min incubation at RT in the dark, flow cytometry analysis was performed to determine cell viability by dye exclusion. Cell populations were gated based on normal light scatter characteristics. Acquisition parameters were set as follows: mean APC‐H channel for fluorescence detection, stop condition 80 µL, flow rate 60 µL/min, core diameter 16 µm, threshold (APC‐H) 100,000.

### 4.12 Migration Assays

For scratch assay cells were grown to confluence on 12‐well plates. The day after seeding, GN11 were treated with 1 and 50 µg/mL PS‐NPs in complete medium for 24 h. Three replicates of each treatment were assayed. Untreated cells were used as negative and positive controls. After treatment, a slit was generated, and medium was replaced: complete medium for untreated (positive control, CTRL) and treated cells; medium without FBS (w/o FBS) for untreated cells (negative controls). Cell migration into the gap was assessed by acquiring phase‐contrast images every 3 h (up to 9 h), using a Zeiss Axiovert 200 inverted microscope equipped with a photometric CoolSnap CDD camera (Roper Scientific) and a Zeiss A‐Plan 10× objective (NA 0.25). For each well, the same area was photographed, positioned above the intersection of the two scratches. The wound area was quantified for each image and was compared to the initial wound area (time t0), using ImageJ software. Three different independent experiments were performed. For Boyden Chamber assays GN11 cells were seeded in 57 cm^2^ plates at a cell density of 1 × 10^6^ cells/plate and treated, 24 h before the assay, with 1 and 50 µg/mL PS‐NPs in complete medium. Untreated cells were used as a positive control (CTRL). Unlike scratch assays, this method exposes cells to a chemotactic gradient, stimulating directed chemotaxis. Rather than gap closure over time, cell migration is quantified by counting the number of migrated cells through a porous membrane (8 µm diameter; NeuroProbe, Gaithersburg, MD, USA; cat. PFB8). Sub‐confluent cells were collected and resuspended in DMEM 0.1% BSA at a final concentration of 2 × 10^6^ cells/mL and exposed to DMEM w/o FBS (negative control) or with 10% of FBS (chemoattractant) for 3 h at 37°C. Unmigrated cells were gently scraped away, and migrated cells were fixed, stained, and mounted onto glass slides as previously described [103]. Each condition was performed in quadruplicate in three independent experiments. For quantitative analysis, the membranes were observed using a Zeiss Axioskop 2 plus brightfield microscope and a Zeiss Plan‐NEOFLUAR 20× objective (NA 0.5). Three random fields were counted for each well/condition, and the mean number of migrating cells/fields for each experimental condition was calculated. Adobe Photoshop 2023 software was used to prepare the presented images.

### 4.13 RNA Sequencing

Total RNA was extracted from frozen cell pellet samples using Qiagen RNeasy Mini kit following manufacturer's instructions (Qiagen, Hilden, Germany; cat. 74104). RNA samples were quantified using Qubit 4.0 Fluorometer (Life Technologies, Monza, Italy) and RNA integrity was checked with Agilent 5300 Fragment Analyzer (Agilent, Palo Alto, CA, USA). RNA‐seq libraries were prepared using the NEB Next Ultra II RNA Library Prep Kit for Illumina following manufacturer's instructions (NEB, Ipswich, MA, USA; cat. E7770). Briefly, mRNAs were first enriched with Oligo(dT) beads. Enriched mRNAs were fragmented according to manufacturer's instruction. First strand and second strand cDNAs were subsequently synthesized. cDNA fragments were end repaired and adenylated at 3’ends, and universal adapters were ligated to cDNA fragments, followed by index addition and library enrichment by limited‐cycle PCR. Sequencing libraries were validated using NGS Kit on the Agilent 5300 Fragment Analyzer (Agilent, Palo Alto, CA, USA) and quantified by using Qubit 4.0 Fluorometer (Invitrogen, Carlsbad, CA, USA). The sequencing libraries were multiplexed and loaded on the flow cell on the Illumina NovaSeq 6000 instrument according to manufacturer's instructions (Illumina, San Diego, CA, USA). The samples were sequenced using a 2 × 150 Pair‐End configuration v1.5. Image analysis and base calling were conducted by the NovaSeq Control Software (v1.7; Illumina, San Diego, CA, USA) on the NovaSeq instrument.

### 4.14 Data Processing and Visualization

Raw sequence data (.bcl files) generated from Illumina NovaSeq were converted into fastq files and de‐multiplexed using Illumina bcl2fastq program version 2.20. One mismatch was allowed for index sequence identification. After investigating the quality of the raw data, sequence reads were trimmed to remove possible adapter sequences and nucleotides with poor quality using Trimmomatic v.0.36. The trimmed reads were mapped to the *Mus musculus* reference genome available on ENSEMBL using the STAR aligner v.2.5.2b. BAM files were generated as a result of this step. Unique gene hit counts were calculated by using *featureCounts* from the Subread package v.1.5.2. Only unique reads that fell within exon regions were counted. After the extraction of gene hit counts, the gene hit counts table was used for downstream differential expression analysis. Using DESeq2, a comparison of gene expression between the groups of samples was performed. The Wald test was used to generate *p*‐values and *l*og2 fold changes (log2FC). Genes with adjusted *p*‐values < 0.05 and absolute |log2FC| >1 were called as differentially expressed genes (DEGs) for each comparison. Functional enrichment analysis was computed on DEGs using reString software (v.0.1.21), while gene prioritization was conducted with ToppGene [47, 104]. Data visualization was performed as previously done with matplotlib and seaborn libraries for Python programming language (v.3.8.5) [105, 106, 107].

### 4.15 Ethics

Patient samples were derived from the Genetic Factors Affecting the Timing of Puberty clinical research network study (IRAS 95781). Ethical approval for human studies was granted by the UK London‐Chelsea NRES committee (13/LO/0257) and by the Ethics Committee of Hospital das Clínicas da Universidade de São Paulo (registration number 37868114.3.0000.0068). All participants provided written informed consent prior to study participation. The study was conducted in accordance with the guidelines of The Declaration of Helsinki. Participants did not receive compensation. The cohort includes 550 participants with disordered puberty recruited from twelve UK participating centers, including 370 probands and relatives with delayed or absent puberty (Male = 212, Female = 158). Participant age is 0–52 years of age inclusive. The Brazilian cohort comprised 77 participants (66 males and 11 females) with self‐limited growth disorders and delayed puberty, evaluated at a specialized growth disorder center. These participants are part of the Delayed Puberty Genetics Consortium patient cohort.

### 4.16 Human Genomic Sequencing

For the GD cohort, whole exome sequencing was performed on DNA extracted from peripheral blood leukocytes, using an Agilent V5 platform (Agilent, Palo Alto, CA, USA) and Illumina HiSeq 2000 sequencing (Illumina, San Diego, CA, USA). Alignment and variant calling were done via a standard GATK pipeline as described previously [108]. Analysis of the called variants was performed using Ingenuity Variant Analysis (Qiagen, www.qiagen.com/ingenuity). Filtering for potential causal variants was carried out using filters for quality control (read depth and Phred strand bias), minor allele frequency (MAF < 0.5% in the ExAC and Genome Aggregation Database (gnomAD) databases v2.0.2), predicted functional annotation (Poly‐Phen, SIFT, REVEL, CADD score) and conservation score (GERP). Potential causal variants were confirmed by Sanger sequencing. For the constitutional delay of puberty cohort, exome sequencing was performed using the Illumina Rapid Capture Exome Kit (Illumina, San Diego, CA, USA) and automated with the Agilent Bravo system (Palo Alto, CA, USA). Sequencing was conducted on HiSeq 2000/2500 platform (Illumina, San Diego, CA, USA). For variant calling, we used Freebayes as previously described [109].

### 4.17 Statistical Analysis

Statistical analysis was performed through One‐Way analysis of variance (ANOVA) or Two‐way ANOVA followed by Dunnett's multiple comparisons test using the Prism 9 software (v.9.5.0; GraphPad Software, San Diego, CA, USA), as specified in figure legends. All experiments were repeated at least three times. Data are presented as mean ± SD, and results were considered significant with a *p*‐value less than 0.05.

## Conflicts of Interest

The authors declare no conflicts of interest.

## Supporting information




**Supporting File**: smll72262‐sup‐0001‐SuppMat.docx.

## Data Availability

The data that support the findings of this study are available from the corresponding authors upon reasonable request. RNA sequencing data are available in Gene Expression Omnibus at https://www.ncbi.nlm.nih.gov/geo/, reference number GSE295898.
