# Supplementary material for: Nanoplastics Impair GnRH Neuron Migration and Neuroendocrine Function: Emerging Players in the Pathogenesis of Reproductive Disorders
Source: Small. 2026 Feb 6;22(15):e06171. doi: 10.1002/smll.202506171 (PMC12980468; doi:10.1002/smll.202506171)
Supplement: Supplementary file 1 — Supporting File: smll72262‐sup‐0001‐SuppMat.docx. [file SMLL-22-e06171-s001.docx]

Supporting Information

**Nanoplastics impair GnRH neuron migration and neuroendocrine function: emerging players in the pathogenesis of reproductive disorders**

*Federica Amoruso^¶^, Alyssa Julia Jennifer Paganoni^¶^, Astrid Saraceni, Andrea Magnani, Alessia Brossa, Giorgio Roberto Merlo, Cristina Matei, Ruben Willemsen, Raíssa Carneiro Rezende, Alexander Augusto de Lima Jorge, Federica Dal Bello, Patrizia Bovolin, Sasha Rose Howard, Roberto Oleari*, Anna Cariboni**


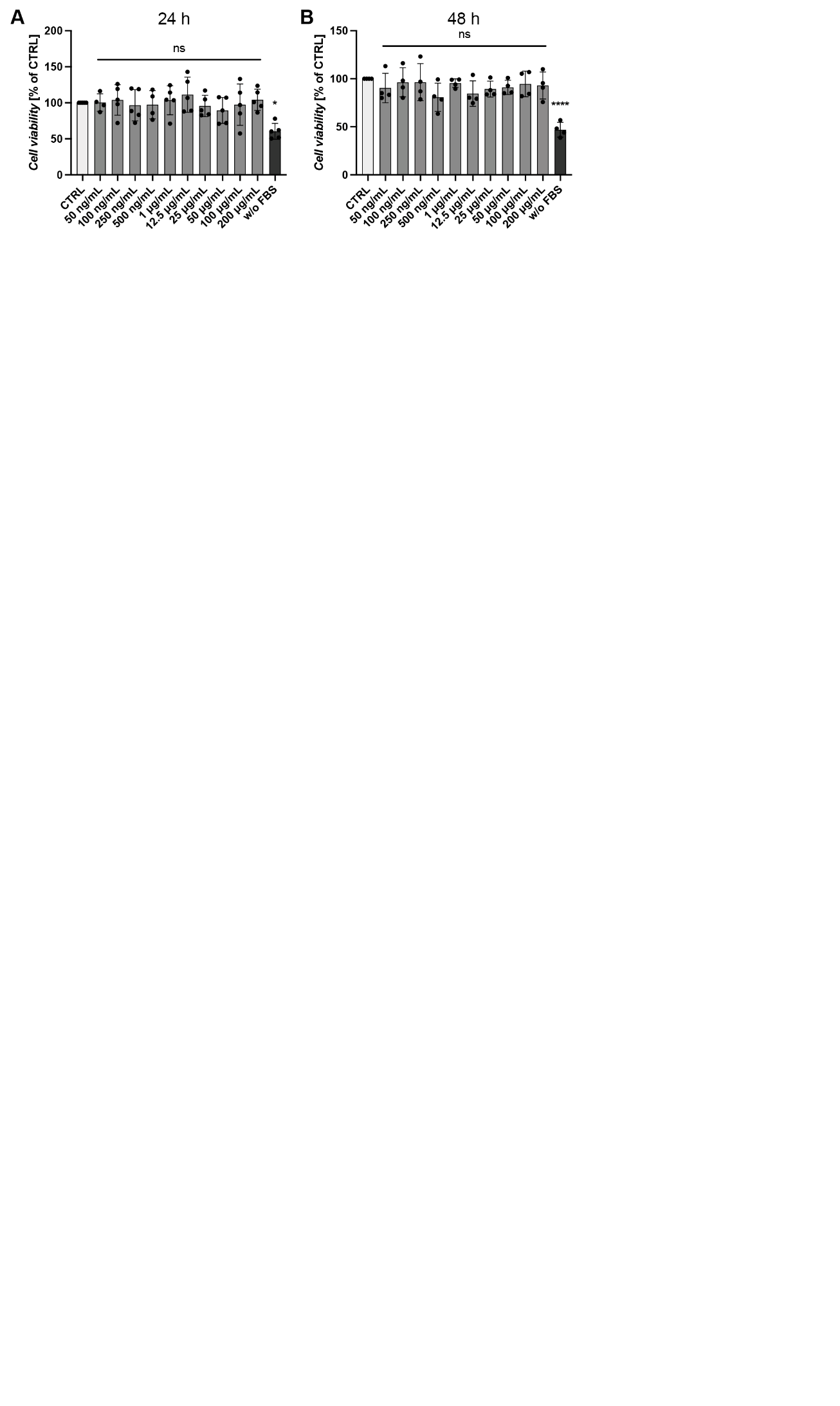


**Figure S1.** (A-B) MTT assays of GT1-7 cells treated for 24 h (A) and 48 h (B) with 500 nm PS-NPs concentrations ranging from 50 ng/mL to 200 μg/mL (*N* = 4; One Way ANOVA followed by Dunnet's multiple comparison test, **p*<0.05; *****p*<0.0001; ns, non significant). Untreated GT1-7 cells grown under normal cell culture conditions were used as positive control (CTRL), while GT1-7 cells grown in the absence of FBS were used as negative control (w/o FBS).


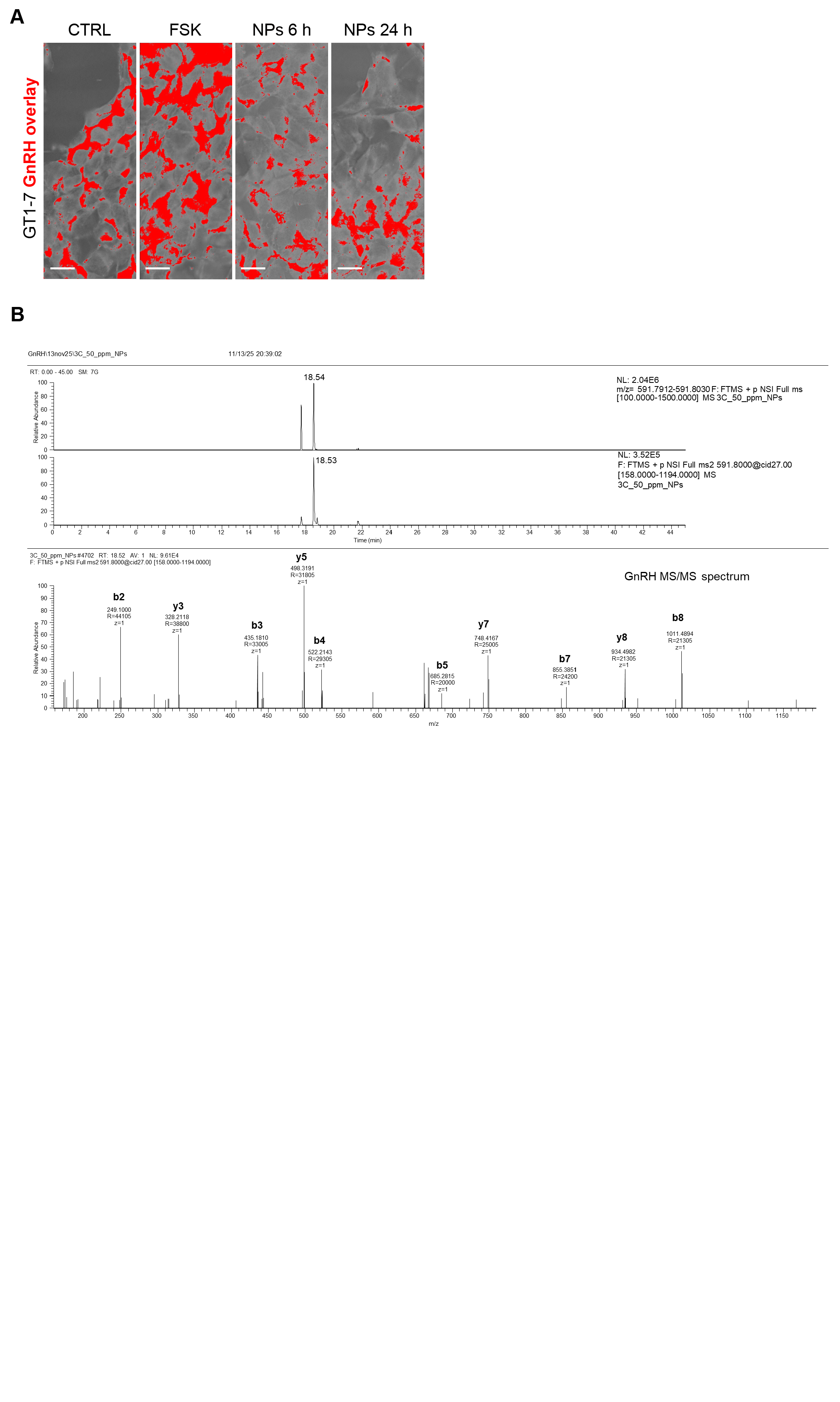


**Figure S2.** (A) GnRH immunostaining of GT1-7 cells treated with 1 μg/mL and 50 μg/mL PS-NPs for 6 and 24 h. Forskolin (FSK) was used as a positive control for GnRH stimulation; untreated GT1-7 cells were used as CTRL. Representative images show a decrease in GnRH staining (red pixels) following PS-NP exposure. Scale bar: 250 μm. (B) Representative image of chromatographic separation and MS/MS spectrum of GnRH in GT1-7 cells exposed to 50 µg/mL PS-NPs for 24 h. The double charged precursor ion of GnRH (*m/z* 591.7) was selected for the MS/MS CID fragmentation. Tandem mass spectrum shows the main product ions of GnRH. Like others peptide, GnRH fragments through peptide-bond cleavage, producing the so-called Biemann ions (y and b ions). The most abundant Biemann ions observed for GnRH are: y5 (m/z = 498.3191), y7 (*m/z* = 748.4167), and b8 (*m/z* = 1011.4894).


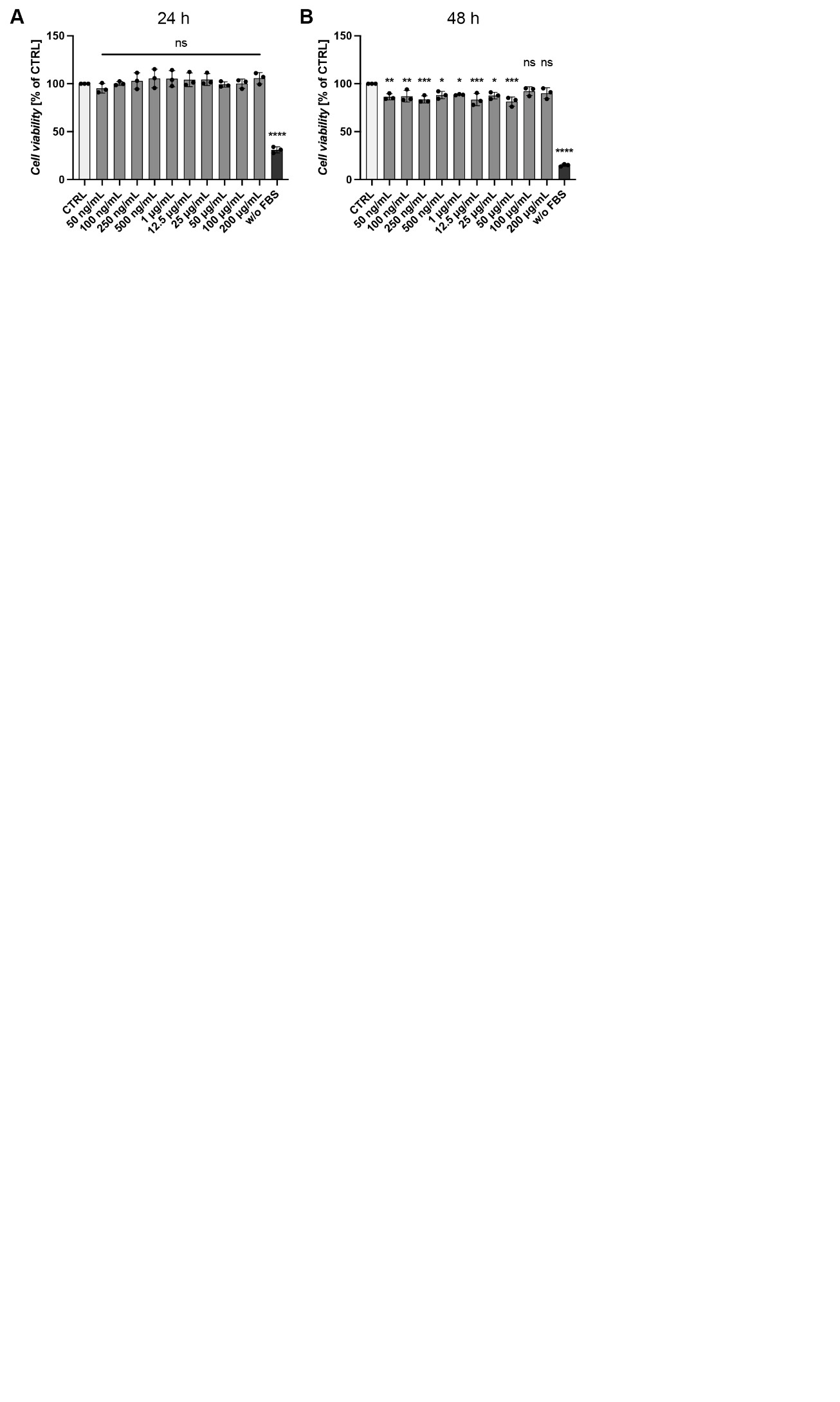


**Figure S3.** (A-B) MTT assays of GN11 cells treated for 24 h (A) and 48 h (B) with 500 nm PS-NPs concentrations ranging from 50 ng/mL to 200 μg/mL (*N* = 3; One Way ANOVA followed by Dunnet's multiple comparison test, **p*<0.05; ***p*<0.01; ****p*<0.001; *****p*<0.0001; ns, non significant). Untreated GN11 cells grown under normal cell culture conditions were used as positive control (CTRL), while GN11 cells grown in the absence of FBS were used as negative control (w/o FBS).


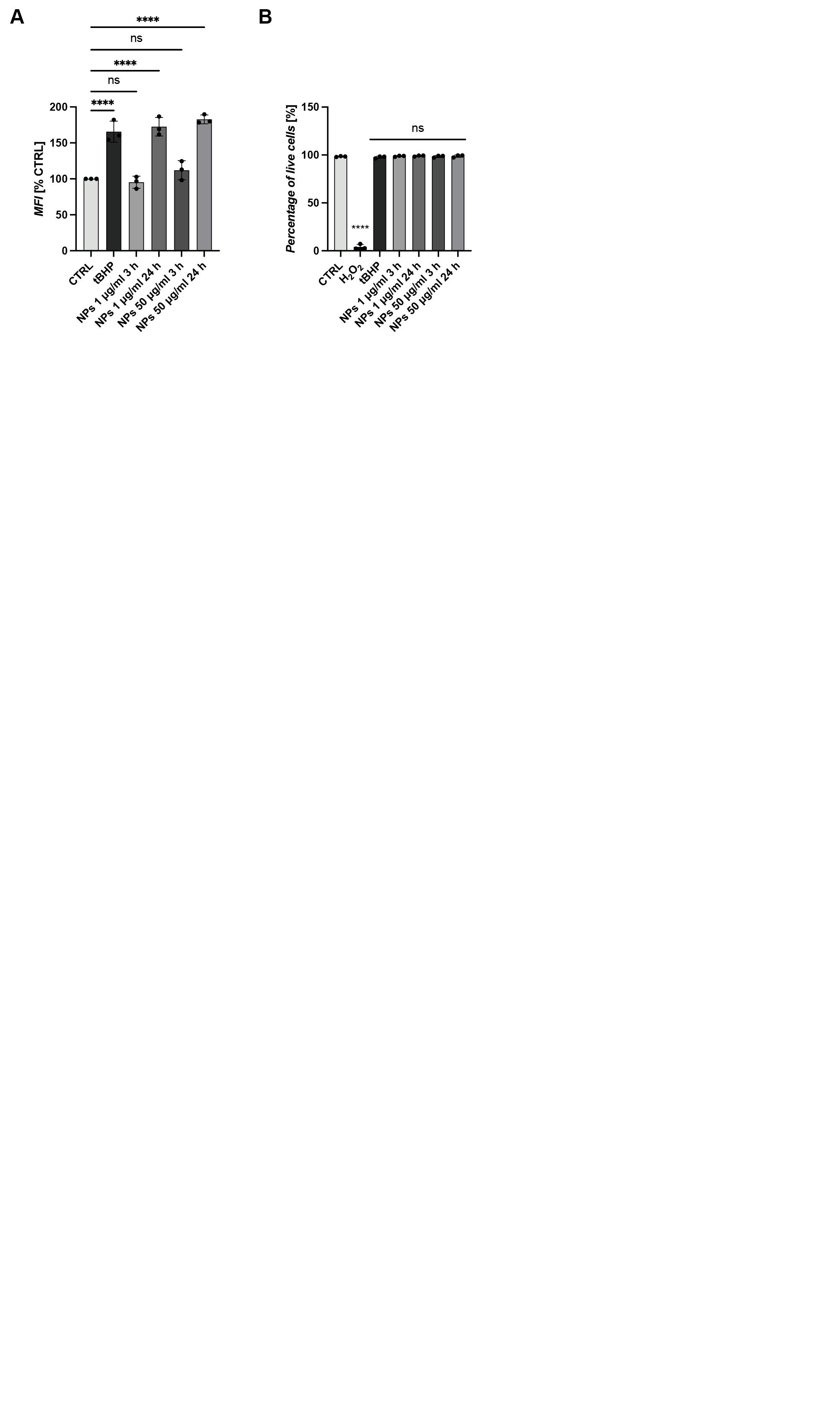


**Figure S4.** (A) Exposure of GN11 cells to 1 μg/mL and 50 μg/mL PS-NPs for 24 results in a significant increase in intracellular ROS levels, indicating a generic stress response (*N* = 3; One Way ANOVA followed by Dunnet's multiple comparison test, *****p*<0.0001; ns, non significant). Untreated GN11 cells served as CTRL, and tert-butyl hydroperoxide (tBHP) was used as positive control for ROS induction. (B) PS-NPs exposure does not increase cell death in GN11 cells. No statistically significant differences were observed in the percentage of live cells (% of total cell number) between untreated cells (CTRL) and those exposed to 1 μg/mL and 50 μg/mL PS-NPs for 3 h or 24 h (*N* = 3; One Way ANOVA followed by Dunnet's multiple comparison test, *****p*<0.0001; ns, non significant). tBHP was used as positive control for ROS production, while treatment with H_2_O_2_ 200 μM for 30 min served as positive control for cell death.


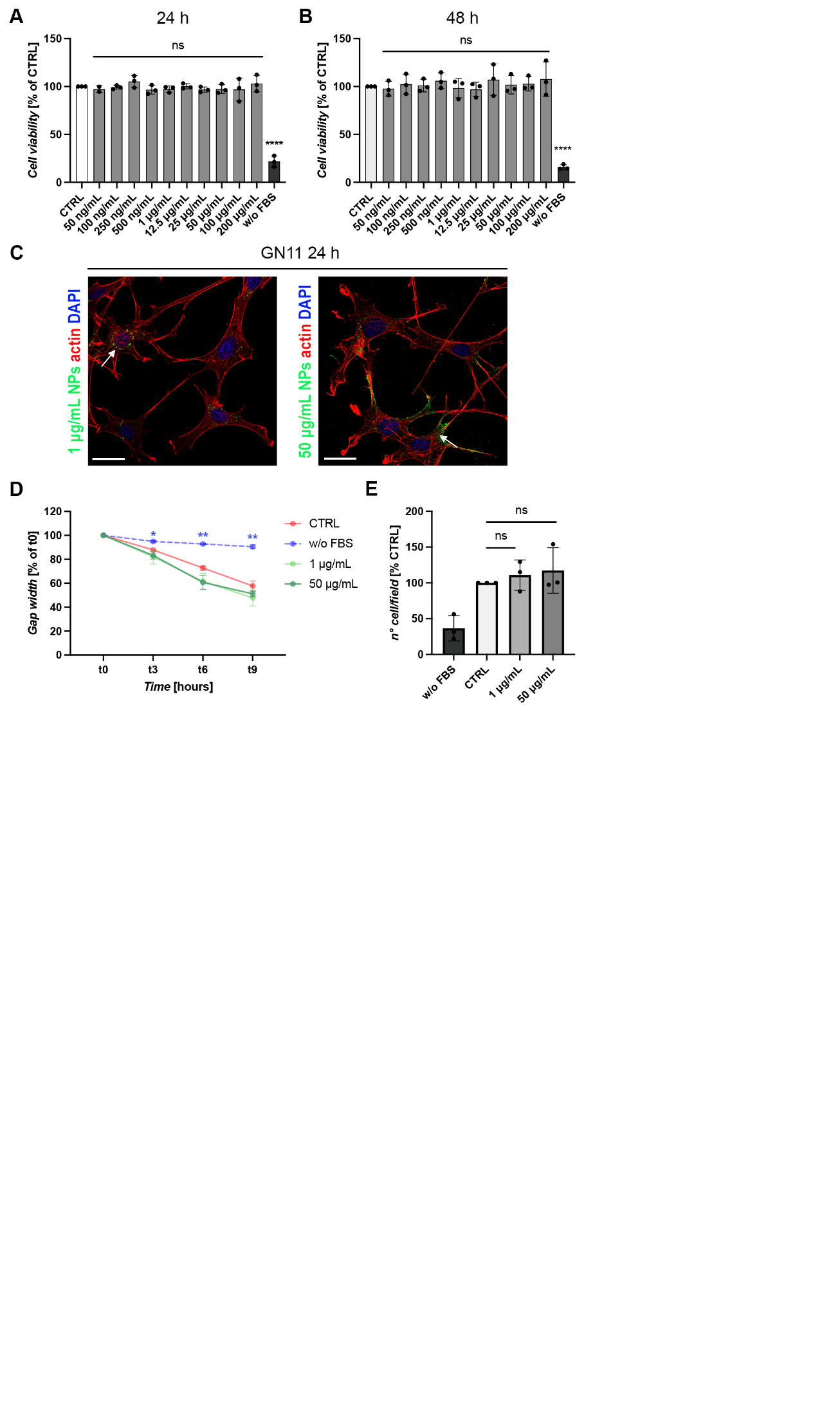


**Figure S5.** (A-B) 50 nm PS-NPs are not cytotoxic to GN11 after 24 and 48 h exposure time. MTT assays of GN11 cells treated for 24 h (A) and 48 h (B) with 50 nm PS-NPs concentrations ranging from 50 ng/mL to 200 μg/mL (*N* = 3; One Way ANOVA followed by Dunnet's multiple comparison test, *****p*<0.0001; ns, non significant). Untreated GN11 cells grown under normal cell culture conditions were used as positive control (CTRL), while GN11 cells grown in the absence of FBS were used as negative control (w/o FBS). (C) Non-toxic concentrations of 50 nm PS-NPs are internalized into GN11 cells. Confocal microscopy images of GN11 cells treated with 1 μg/mL (left panel) and 50 μg/mL (right panel) PS-NPs for 24 h. Fluorescent PS-NPs are shown in green and indicated with arrows, DAPI-labelled nuclei are in blue and phalloidin-labelled cytoskeleton (F-actin) in red. Scale bars: 50 μm. (D-E) 50 nm PS-NPs do not impact GN11 neuron migration. (D) Evaluation of migratory capacity of untreated GN11 cells (CTRL) and GN11 cells treated for 24 h with 1 μg/mL and 50 μg/mL 50 nm PS-NPs towards FBS, assessed by scratch assays. Untreated GN11 cells exposed to DMEM without FBS were used as negative control (w/o FBS). Graph of gap width (% of t0) as a function of time (hours) after wound generation. No reduction in migratory capacity was observed for treated cells respect to CTRL (*N* = 3; 2-way ANOVA followed by Dunnet's multiple comparison test, **p*<0.05; ***p*<0.01). (E) Assessment of GN11 chemomigratory capacity (CTRL; 1 μg/mL and 50 μg/mL PS-NP-treated cells) towards complete DMEM (with FBS) by transwell assay. Migration of untreated cells to DMEM without FBS (w/o FBS) was used as negative control. Graph of the number of migrated cells/field (% of CTRL), showing no statistically significant reduction in cell migration for GN11 cells treated with 50 nm PS-NP concentrations (*N* = 3; One Way ANOVA followed by followed by Dunnet's multiple comparison test; ns, non significant).


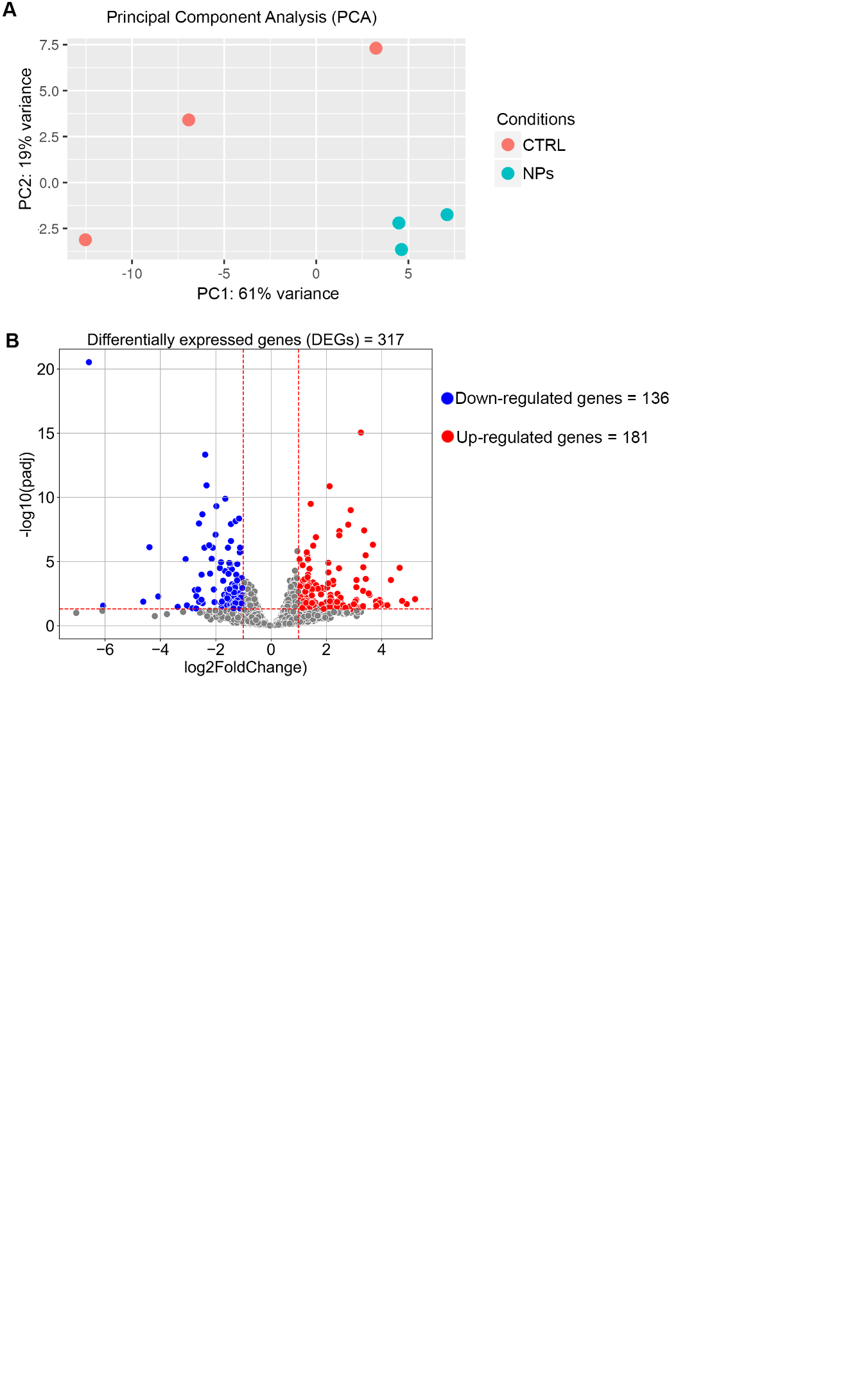


**Figure S6.** (A) Dimensionality reduction was performed on gene expression space for each GN11 CTRL and GN11 NP cell sample, and the first two principal coordinates (PCs) are charted. (B) Volcano plot showing differentially expressed genes (DEGs) (|Log_2_FC| > 1 and adjusted *p*-value < 0.05) between GN11 CTRL and GN11 NP cells. GN11 NPs up-regulated genes are indicated in red, whereas down-regulated genes are in blue; gray dots indicate non-DEGs.

**Table S1.** List of genes used as training set for ToppGene prioritization analysis.

| IGD genes^a)^ | Other genes^b)^ |
| --- | --- |
| AMH | PLXNB1 |
| AMHR2 | SEMA3G |
| ANOS1 | SLIT2 |
| AXL | ROBO3 |
| CCDC141 | PTCH1 |
| CHD7 | MIR200A |
| CHL1 | RMST |
| DCC | JAG1 |
| DUSP6 | CXCR4 |
| FEZF1 | ISL1 |
| FGF8 | ISL2 |
| FGF17 | DBN1 |
| FGFR1 | OTX2 |
| FLRT3 | VAX1 |
| GLI3 | SIX3 |
| HS6ST1 | SIX6 |
| IGSF10 | ARHGAP5 |
| IL17RD | ARHGAP35 |
| NDNF |  |
| NRP1 |  |
| NRP2 |  |
| NSMF |  |
| NTN1 |  |
| OTUD4 |  |
| PLXNA1 |  |
| PLXNA3 |  |
| PROK2 |  |
| PROKR2 |  |
| SMCHD1 |  |
| SEMA3A |  |
| SEMA3E |  |
| SEMA3F |  |
| SEMA7A |  |
| SOX10 |  |
| SPRY4 |  |
| TCF12 |  |
| TUBB3 |  |
| WDR11 |  |

^a)^ Known GD causative genes implicated in GnRH neuron developmental processes according to Oleari et al., 2021 and Cangiano et al., 2020. ^[11,110]^

^b)^ Genes implicated in GnRH neuron biology through *in vivo* studies.

**Table S2.** Clinical and biochemical data of the male proband carrying the rare and predicted deleterious p.R315W variant in NPAS2. *after GnRH stimulation test with intravenous administration of 100ug of GnRH and measurement at 30 minutes post injection. SDS- standard deviation score for age and sex, S-LH – serum luteinizing hormone, S-FSH – serum follicle stimulating hormone, S-T – serum testosterone, TFTS – thyroid function test, IGF1 – insulin-like growth factor 1. Normal ranges for basal concentrations are shown in brackets.

| Age [year] | 17.1 | 17.8 | 18.3 |
| --- | --- | --- | --- |
| Height [cm] | 165.5 | 168.0 (-1.0 SDS) | 172.5 (-0.3 SDS) |
| Weight [kg] | 72.8 | 75.2 | 83.9 |
| BMI [kg/m^2^] | 26.7 (+1.9 SDS) | 26.6 (+1.8 SDS) | 28.2 (+2.0 SDS) |
| Bone age [year] | 13.5 |  |  |
| Testes vol [mL] | 5 mL / 6 mL | 8 mL / 10 mL | 15 mL / 20 mL |
| Genital Tanner stage | G2 | G3 | G5 |
| Pubic hair Tanner stage | P2 | P3 | P4 |
| Axillary hair Tanner stage | A2 | A2 | A3 |
| S-LH [IU/L](1.5-4.9) | 0.8 (peak 16*) | 2.4 | - |
| S-FSH [IU/L] (2.4-6.2) | 2.2 (peak 5*) | 2.4 | - |
| S-T [nmol/L] (8.6-23.4) | 1.0 | 4.1 | - |
| Inhibin B [pg/mL] | - | 145 | - |
| TFTs | Normal |  |  |
| IGF1 [ug/L] (15.5-67) | 31.2 |  |  |
| Cortisol [nmol/L] | 199 |  |  |

**Table S3.** Clinical and biochemical data of the male proband carrying the p.V124I variant in *NPAS2*. SDS- standard deviation score for age and sex, S-LH – serum luteinising hormone, S-FSH – serum follicle stimulating hormone, S-T – serum testosterone, TFTS – thyroid function test, IGF1 – insulin-like growth factor 1. Normal ranges for basal concentrations are shown in brackets.

| Age [year] | 13.6 | 14.5 | 15.3 |
| --- | --- | --- | --- |
| Height [cm] | 135.5 (-3.1 SDS) | 142.8 (-2.8 SDS) | 150.4 (-2.5) |
| Weight [kg] | 28.2 | 33.3 | 37.3 |
| BMI [kg/m2] | 15.4 (-1.9 SDS) | 16.3 (-1.6 SDS) | 16.5 (-1.8) |
| Bone age [year] | 10.0 | 12.5 | 13.5 |
| Testes vol [mL] | NA | 6 mL / 6 mL | NA |
| Genital Tanner stage | G1 | G2 | G3 |
| Pubic hair Tanner stage | P1 | P2 | P3 |
| S-LH [IU/L] | 2.1 | NA | 3.1 |
| S-FSH [IU/L] | 5.9 | NA | 7.3 |
| S-T [nmol/L) | 1.6 | NA | 9.7 |
| TFTs | Normal | Normal | NA |
| IGF1 [ug/L] (15.5-67) | NA | 314 (220 – 972) | NA |
